# Interferon hyperactivity impairs cardiogenesis in Down syndrome via downregulation of canonical Wnt signaling

**DOI:** 10.1016/j.isci.2023.107012

**Published:** 2023-06-05

**Authors:** Congwu Chi, Walter E. Knight, Andrew S. Riching, Zhen Zhang, Roubina Tatavosian, Yonghua Zhuang, Radu Moldovan, Angela L. Rachubinski, Dexiang Gao, Hongyan Xu, Joaquin M. Espinosa, Kunhua Song

**Affiliations:** 1 Division of Cardiology, Department of Medicine, University of Colorado Anschutz Medical Campus; Aurora, CO 80045, USA; 2 Linda Crnic Institute for Down Syndrome, University of Colorado Anschutz Medical Campus; Aurora, CO 80045, USA; 3 Gates Center for Regenerative Medicine and Stem Cell Biology, University of Colorado Anschutz Medical Campus; Aurora, CO 80045, USA; 4 Consortium for Fibrosis Research & Translation, University of Colorado Anschutz Medical Campus; Aurora, CO 80045, USA; 5 Department of Pharmacology, University of Colorado Anschutz Medical Campus; Aurora, CO 80045, USA; 6 Department of Pediatrics, University of Colorado Anschutz Medical Campus; Aurora, CO 80045, USA; 7 Department of Population Health Sciences, Medical College of Georgia, Augusta University; Augusta, GA 30912, USA

**Keywords:** Cell biology, Stem cells research, Developmental biology, Transcriptomics

## Abstract

Congenital heart defects (CHDs) are frequent in children with Down syndrome (DS), caused by trisomy of chromosome 21. However, the underlying mechanisms are poorly understood. Here, using a human-induced pluripotent stem cell (iPSC)-based model and the Dp(16)1Yey/+ (Dp16) mouse model of DS, we identified downregulation of canonical Wnt signaling downstream of increased dosage of interferon (IFN) receptors (IFNRs) genes on chromosome 21 as a causative factor of cardiogenic dysregulation in DS. We differentiated human iPSCs derived from individuals with DS and CHDs, and healthy euploid controls into cardiac cells. We observed that T21 upregulates IFN signaling, downregulates the canonical WNT pathway, and impairs cardiac differentiation. Furthermore, genetic and pharmacological normalization of IFN signaling restored canonical WNT signaling and rescued defects in cardiogenesis in DS *in vitro* and *in vivo*. Our findings provide insights into mechanisms underlying abnormal cardiogenesis in DS, ultimately aiding the development of therapeutic strategies.

## Highlights

• Interferon signaling is hyperactivated during cardiogenesis in Down syndrome
• Canonical Wnt signaling is downregulated during cardiogenesis in Down syndrome
• Interferon hyperactivity impairs cardiogenesis by downregulating the Wnt pathway

## Graphical abstract

**Figure:**
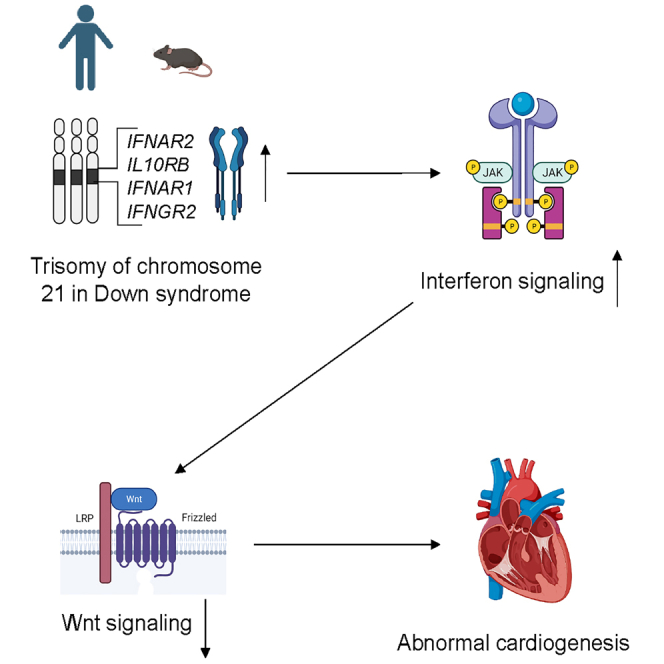

## Introduction

Down syndrome (DS), caused by trisomy of human chromosome 21 (HSA21) (T21), is a leading cause of intellectual and developmental disability, affecting ∼1 in 700 newborns.^1^^,^^2^^,^^3^ Individuals with DS are predisposed to various medical conditions including congenital heart defects (CHDs), autoimmune disorders, autism spectrum disorders, and leukemia.^1^^,^^2^^,^^3^^,^^4^ Approximately 50% of newborns with DS are affected by CHDs including ventricular septal defects (VSD), atrial septal defects (ASD), and atrioventricular septal defects.^5^ CHDs are a primary and significant risk factor for mortality in people with DS through age twenty.^6^^,^^7^^,^^8^ Despite this extremely high incidence of heart defects in DS, the underlying mechanisms are poorly understood.^3^

Intensive effort has been focused on identifying chromosomal regions of HSA21 responsible for CHDs.^9^^,^^10^^,^^11^^,^^12^ HSA21 is homologous to portions of at least three murine chromosomes: MMU16, MMU17, and MMU10 and various mouse models of DS have been created with triplication of these regions and smaller subregions.^13^^,^^14^^,^^15^^,^^16^^,^^17^^,^^18^^,^^19^ Trisomy for MMU10 or MMU17 does not cause CHDs in these mouse models.^16^ However, ∼50% of mice with trisomy of MMU16, such as Dp(16)1Yey/+ (hereafter referred to as Dp16), display CHDs,^17^ resembling cardiac defects in people with DS. Using mouse strains with trisomy of MMU16, Liu et al. suggested a 3.7 Mb genomic region from *Tiam1* to *Kcnj6* was sufficient to cause heart defects in mice.^12^ This 3.7 Mb genomic region contains a cluster of four interferon (IFN) receptor (IFNR) genes: *Ifnar, Ifnar2*, *Ifngr2,* and *Il10rb*. In addition, genetic variants in human *IFNGR2* and *IL10RB* have been associated with CHDs in DS from two cohort studies composed of 198 individuals and 702 individuals, respectively.^20^^,^^21^ In the Dp16 mouse model, we recently showed that normalization of copy number of the *Ifnr* cluster prevented heart malformations,^22^ but the mechanisms by which hyperactive IFN signaling may disrupt normal heart development await elucidation.

Studies in mouse and human pluripotent stem cells have established a hierarchical model of cardiac lineage differentiation and specification of cardiac progenitors precisely regulated by specific signaling pathways.^23^^,^^24^^,^^25^^,^^26^^,^^27^^,^^28^ Among them, activation of canonical Wnt/β-catenin pathway is required for expansion and differentiation of cardiac progenitor cells (CPCs) *in vitro* and *in vivo.*^29^^,^^30^^,^^31^^,^^32^^,^^33^^,^^34^^,^^35^^,^^36^^,^^37^ However, a potential role for dysregulation of the canonical Wnt pathway in cardiogenesis in DS has not been defined.

Since genes contained on HSA21 are spread over three syntenic regions on MMU16, MMU17, and MMU10, it is difficult to create a mouse model to fully recapitulate the effects of T21. Recently, human models using induced pluripotent stem cells (iPSCs) derived from individuals with DS have emerged to study pathogenesis of various medical conditions caused by T21.^38^^,^^39^^,^^40^^,^^41^^,^^42^^,^^43^ Bosman et al. generated one line of human embryonic stem cells (hESC) from a donated embryo with T21, and two control hESC lines from siblings, and another hESC line from an unrelated embryo with T21.^43^ These four hESC lines were differentiated into embryoid bodies (EBs) and gene expression in EBs was measured at various differentiation stages by real-time PCR (qPCR) and bulk RNA-sequencing (RNA-seq). This study identified hundreds of genes that were dysregulated in cardiomyocyte clusters differentiated from T21 hESC lines. However, the mechanisms driving such dysregulated cardiac expression programs were not elucidated in this report.^43^

Here, using a combination of a human iPSC-based model and Dp16 mice, we demonstrate that hyperactivation of IFN signaling downstream of increased dosage of IFNR genes downregulates canonical Wnt signaling and leads to cardiogenic dysregulation in DS. We differentiated human iPSCs derived from individuals with DS and co-occurring CHDs (DS/CHD iPSCs), as well as from healthy donors (control iPSCs) into cardiac cells. Increased IFN signaling, decreased canonical Wnt pathway, and impaired cardiac differentiation were observed during differentiation of DS/CHD iPSCs. Binding to IFN ligands to IFNRs induces JAK/STAT signaling, and markers of elevated JAK/STAT signaling were observed in DS/CHD iPSCs at the protein and transcriptome levels. Normalization of IFN signaling with a JAK inhibitor (JAKi) restored canonical Wnt signaling and rescued defects in cardiac differentiation of DS/CHD iPSCs. JAKi treatment normalized the canonical Wnt pathway and prevented heart malformations in Dp16 embryos. These data from both *in vitro* and *in vivo* studies define a role for a signaling axis involving hyperactive IFN signaling and decreased Wnt signaling in cardiogenic defects in DS, while also revealing avenues for potential therapeutics.

## Results

### Trisomy 21 impairs cardiac differentiation of iPSCs

To characterize cardiogenesis in DS, we derived iPSCs from three individuals with DS and co-occurring CHDs (DS/CHD iPSCs), and from three healthy donors (control iPSCs) using non-integrating modified mRNAs and miRNAs^44^ (Table S1). T21 in individual iPSC lines was confirmed by karyotyping (Table S1 and Figure S1A). Pluripotency of these human iPSC lines was validated by assessing the expression of pluripotent stem cell markers (Figure S1B). To prevent the reversion of T21 to disomy of chromosome 21 (D21), we performed all experiments using iPSCs undergoing less than 20 passages. Karyotypic analysis of individual iPSC lines with the highest passage numbers in this study demonstrated correct copy numbers of chromosome 21 in these iPSC lines (Figure S1A and Table S1).

**Figure 1:**
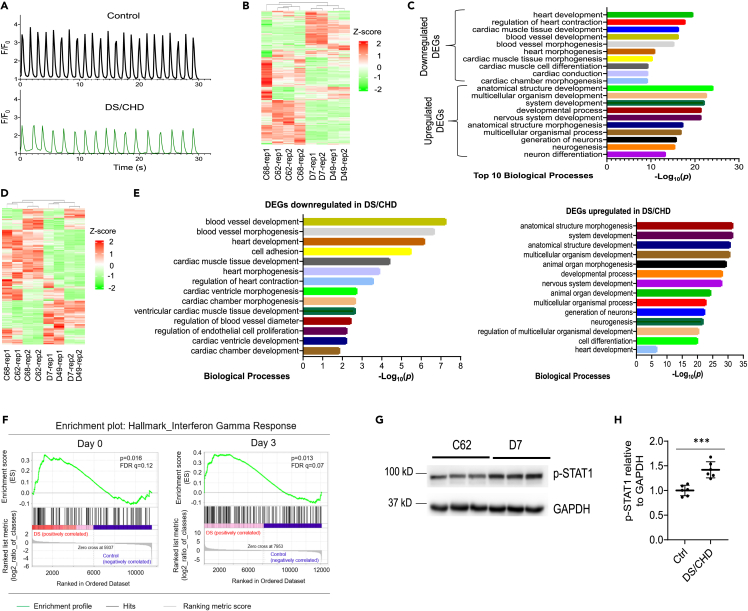
Trisomy 21 causes hyperactivation of IFN signaling and impairs cardiac differentiation *in vitro* (A) Representative control (C68) and DS/CHD (D49) Ca^2+^ transient traces measured with overexpressed GCaMP6f in iPSC-CMs on day 13 of cardiac differentiation. Florescence intensity was normalized to baseline (F/F_0_). Quantification of Ca^2+^ transient amplitude and frequency is shown in Figure S3. (B) Heatmap of gene expression as assayed by RNA-seq in control and DS/CHD cells on differentiation day 7. Two replicates (rep1 and rep2) were used for each iPSC line for RNA-seq. (C) Top GO terms associated with significantly up- and downregulated genes with a >1.5-fold change in DS/CHD cells on differentiation day 7, compared with control. (D) Heatmap of gene expression as assayed by RNA-seq in control and DS/CHD cells on differentiation day 13. Two replicates (rep1 and rep2) were used for each iPSC line for RNA-seq. (E) GO analysis of significantly up- and downregulated genes with a >1.5-fold change in DS/CHD cells on differentiation day 13, compared with control. (F) Gene set enrichment analysis (GSEA) of RNA-seq on day 0 and day 3 showing that genes upregulated in cells differentiated from DS/CHD iPSCs were enriched in IFN gamma signaling response. RNA-seq was performed on C62, C68, D7, and D49 iPSC lines. Genes with expression ≥25 FPKM were selected for analysis. Genes preferentially expressed in DS/CHD cells are shown in Figures S4A and S4B. (G and H) Immunoblotting analysis of phosphorylated STAT1(Tyr701) (p-STAT1) and GAPDH in cells differentiated from control (Ctrl) (C62 and C68) and DS/CHD (D7 and D49) iPSCs on differentiation day 3. Representative western blots are shown in G. Quantification of relative p-STAT1 levels are presented in H. Each filled circle represents one independent experiments for each iPSC line. Three independent inductions were performed for each line. Data are presented as mean ± SD. ∗∗∗p < 0.001, unpaired Student’s *t* test.

Next, we investigated whether human D21 iPSCs could model cardiac lineage differentiation by analyzing expression of lineage-specific markers using a monolayer-based protocol as shown previously^45^^,^^46^^,^^47^ (Figure S2A). RNA-seq analysis of control iPSC lines (C62 and C68) indicated that all cardiac lineage markers were absent in undifferentiated iPSCs. In contrast, MESP1, a pre-cardiac marker, reached its highest expression at differentiation day 3. ISL1 and NKX2-5, markers for cardiac progenitors, reached peak expression at days 5–7. Markers for cardiac lineages including endothelial cells, smooth muscle cells, and cardiomyocytes were activated beginning on day 7 (Figure S2B). We next investigated whether DS/CHD iPSCs exhibit defects during cardiac differentiation. We induced cardiac differentiation on two control lines (C62 and C68) and two DS/CHD lines (D7 and D49) (Table S1), and monitored differentiation efficiency by qPCR analysis of *NKX2-5* and *ISL1* expression at day 7. Expression of these cardiac progenitor markers was significantly downregulated in DS/CHD cells, compared to control cells (Figure S2C). We specifically examined cardiac differentiation by observing the initiation of spontaneous beating. Spontaneous beating clusters in control iPSC cultures appeared on day 9 ± 1 post-induction. However, spontaneous beating clusters in DS/CHD iPSC cultures did not appear until day 12 ± 1 post-induction. Weaker and slower calcium transients were also observed in beating clusters differentiated from DS/CHD iPSCs, compared to controls (Figures 1A and S3), consistent with the finding that beating clusters in DS/CHD iPSC cultures exhibited weak and slow contraction (Videos S1 and S2). Next, we profiled global gene expression using RNA-seq in control lines (C62 and C68) and paired DS/CHD lines (D7 and D49) on differentiation day 7, when cardiac progenitors begin to differentiate into cardiac lineages. Unsupervised clustering analysis demonstrated that the two controls and two DS/CHD lines grouped together, indicating similar biological characteristics within the same karyotype, whereas the gene expression in control cultures was distinct from DS/CHD cultures (Figure 1B). We then performed Gene Ontology (GO) analysis on differentially expressed genes (DEGs) between control and DS/CHD cells. Top GO terms associated with downregulated DEGs in DS/CHD cells involved cardiac differentiation and heart development (Figure 1C). In contrast, upregulated genes in DS/CHD cells were generally associated with non-cardiac development, such as nervous system development (Figure 1C). Notably, we observed maximal numbers of contractile clusters in control and DS/CHD cultures on differentiation day 13. Therefore, we analyzed late cardiogenesis in iPSC-derived cells on day 13. Similar to transcriptomic results on day 7, RNA-seq demonstrated that patterns of gene expression in control cultures (C62 and C68) were distinct from DS/CHD cultures (D7 and D49) on differentiation day 13 (Figure 1D). GO analysis of the DEGs demonstrated that downregulated DEGs in DS/CHD cells are predominantly associated with heart development events (Figure 1E). Upregulated DEGs in DS/CHD cells are predominantly associated with non-cardiac development, such as neurogenesis (Figure 1E). We also observed some upregulated DEGs associated with cardiac development (Figure 1E). Taken together, these data indicate that T21 dysregulates cardiogenic gene expression along with defects in cardiac differentiation of DS/CHD iPSCs.

Video S1. Spontaneous contraction of clusters in cultures differentiated from control iPSCs (C62) on day 13 post-induction of differentiation, related to Figure 1

Video S2. Spontaneous contraction of clusters in cultures differentiated from DS/CHD iPSCs (D7) on day 13 post-induction of differentiation, related to Figure 1

### T21 induces hyperactive IFN signaling during cardiac cell differentiation

We then sought to identify specific genes on HSA21 and signaling pathways that could contribute to impaired cardiogenesis in the iPSC model. Given that impaired cardiac differentiation of DS/CHD iPSCs was observed on day 7 (Figures 1B and 1C), we hypothesized that dysregulation of signaling pathways that cause defects in cardiac differentiation must occur at earlier time points. We performed gene set enrichment analysis (GSEA)^48^^,^^49^ of RNA-seq data generated on day 0 and day 3. GSEA demonstrated that genes upregulated in DS/CHD cells were enriched in IFN-inducible genes (ISGs) (Table S2, Figures 1F, S4A, and S4B). qPCR analysis showed that IFNRs encoded on HSA21, especially the type I receptors *IFNAR1* and *IFNAR2*, were significantly upregulated in DS/CHD cells during early cardiac differentiation of iPSCs (Figure S4C). Recently, hyperactivation of IFN signaling was consistently observed in multiple immune and non-immune cell types from people with DS.^50^^,^^51^^,^^52^^,^^53^ Additionally, genomic sequencing suggested that genetic variants in HSA21 genes encoding IFNRs may be associated with CHDs in DS.^20^^,^^21^ Furthermore, in the Dp16 mouse model, normalization of IFNR copy number prevented heart malformations.^22^ Importantly, Dp16 embryonic hearts display transcriptome changes indicative of IFN hyperactivity that are dependent on IFNR triplication.^22^ However, it is unclear whether hyperactivation of IFN signaling also occurs during human cardiogenesis in DS. The IFN signaling cascade is comprised of three combinations of receptors and downstream effectors.^54^ Binding of diverse IFNs to specific receptors activates tyrosine kinase 2, Janus kinase 1 (JAK1), and/or JAK2, which results in phosphorylation, dimerization, and nuclear translocation of signal transducer and activator of transcription (STAT) factors. Immunoblotting analysis demonstrated that phosphorylation of STAT1 (p-STAT1) was significantly upregulated on day 3 when DS/CHD iPSCs lost pluripotency and began to differentiate into MESP1^+^ cardiovascular progenitor cells (Figure S2B and Figures 1G and 1H). Taken together, these results demonstrate that IFN signaling is hyperactivated during cardiac differentiation in DS/CHD iPSCs.

### Inhibition of IFN signaling improves cardiac differentiation of DS/CHD iPSCs

**Figure 2:**
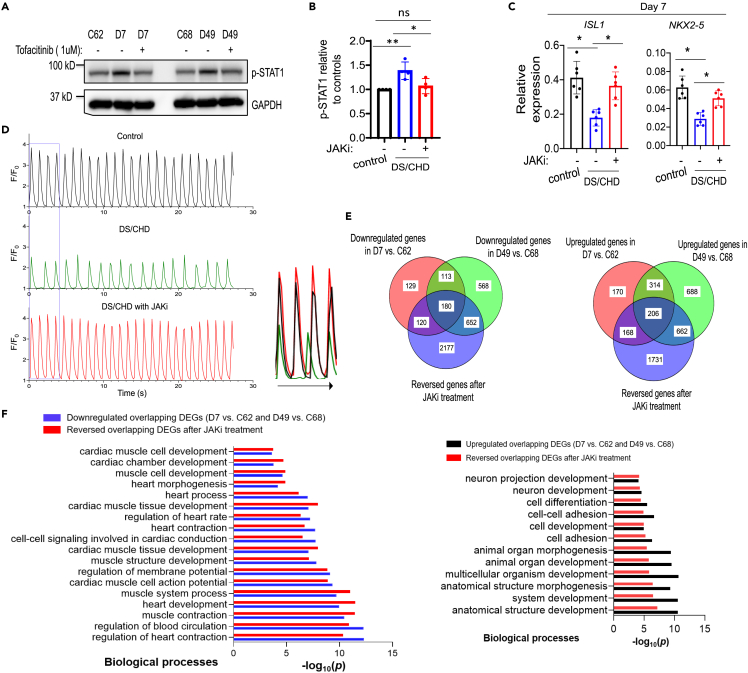
Reduction of the activity of IFN signaling pathways reverses defects in cardiac differentiation of DS/CHD iPSCs (A and B) Immunoblotting analysis of p-STAT1 and GAPDH on differentiation day 3. DS/CHD iPSCs were treated with DMSO or 1 μM JAKi from day 0 to day 3. A representative immunoblotting image is shown in A. Quantification of relative p-STAT1 levels in control cells (C62 and C68) and DS/CHD cells (D7 and D48) on differentiation day 3 in B. Each filled circle represents one independent induction, with two independent experiments for each line. Data are presented as mean ± SD. ∗p < 0.05, ∗∗p < 0.01, ns, not significant, ordinary one-way ANOVA. (C) qPCR analysis of gene expression in iPSC-derived cells on differentiation day 7. DS/CHD iPSCs were treated with 1.0 μM JAKi beginning on day 0. Each filled circle represents one independent induction, with three independent inductions for each cell line. Control lines are C62 and C68 and DS/CHD lines are D7 and D49. Data are presented as mean ± SD. ∗p < 0.05, Ordinary one-way ANOVA. (D) Representative traces of normalized GCaMP6f signal intensity showing Ca^2+^ transients in control iPSC-CMs (C68), DS/CHD iPSC-CMs (D49), and DS/CHD iPSC-CMs treated with the JAKi from day 0 to day 3. Ca^2+^ signals were imaged on day 13. Merged traces in the box are shown on right. Quantification of Ca^2+^ transient amplitude and frequency is shown in Figure S3. (E and F) Transcriptomic analysis using RNA-seq. Three independent experiments were performed for each iPSC line (C62, C68, D7, and D48) under each condition for RNA-seq. FPKM ≥30 was set as cutoff to filter transcripts. Fold change ≥1.5 and p < 0.05 were set as criteria to determine DEGs. Venn diagrams in E demonstrating numbers of genes whose dysregulation was rescued by upon JAKi treatment on differentiation day 13. GO analysis in F of the overlapping downregulated and upregulated DEGs in DS/CHD-differentiated cells shown in E on day 13 and the rescue effects of the JAKi.

Next, we sought to determine whether inhibition of IFN signaling was sufficient to rescue defects in human cardiogenesis *in vitro*. First, we normalized IFN receptor levels using shRNAs to knockdown specific receptors. We first focused on *IFNAR1* and *IFNAR2* because their expression in DS/CHD iPSC-derived cells increased more than *IFNGR2* and *IL10RB*, compared to healthy euploid controls (Figure S4C). After we transduced iPSC lines with either scramble shRNA or shRNAs targeting *IFNAR1* and *IFNAR2*, we selected stable iPSC lines wherein the expression of *IFNAR1* and *IFNAR2* was comparable to control cells (Figure S5A). Normalization of *IFNAR1* and *IFNAR2* levels in DS/CHD iPSCs decreased the activity of IFN signaling by decreasing p-STAT1 levels (Figure S5B) and rescued defects in cardiac differentiation by increasing cardiac gene expression (Figures S5B and S5C). We next normalized IFN-JAK signaling by treating differentiating DS/CHD iPSCs with 1 μM of tofacitinib, a JAK1/3 inhibitor (hereafter referred to as JAKi). This dose of JAKi normalized the activity of the IFN-JAK-STAT pathway in DS/CHD iPSC-derived cells, as assessed by immunoblotting analysis of p-STAT1 levels (Figures S6A, 2A, and 2B). Treatment with JAKi also rescued defects in cardiac differentiation of DS/CHD iPSCs by increasing cardiac gene expression by qPCR (Figure 2C), resulting in increasing numbers and strength of contractile clusters (Videos S3, S4, and S5). Spontaneously beating clusters appeared on differentiation day 9.5 in control iPSC cultures, but until differentiation day 11.5 in DS/CHD iPSC cultures. However, spontaneously beating clusters appeared in DS/CHD iPSC cultures treated with JAKi on differentiation day 9.8, comparable to control cultures (Figure S6B). JAKi treatment also increased frequency and strength of Ca^2+^ transients of DS/CHD beating clusters to the level of controls (Figures 2D and S3A, and S3B). To further investigate molecular mechanisms by which inhibition of IFN signaling restored cardiac differentiation of DS/CHD iPSCs, we performed RNA-seq analysis to profile global gene expression in cells differentiated from two pairs of iPSCs (C62 versus D7 and C68 versus D49) on differentiation day 13. RNA-seq analysis revealed that JAKi treatment normalized many DEGs between control and DS/CHD cultures. The expression of 374 of 858 upregulated DEGs and 300 of 542 downregulated DEGs in D7 vs. C62, and 868 of 1870 upregulated DEGs and 832 of 1513 downregulated DEGs in D49 vs. C68, was effectively reversed by JAKi treatment. A Venn diagram analysis revealed that the expression of 206 upregulated overlapping DEGs and 180 downregulated overlapping DEGs between paired lines was reversed by JAKi (Figure 2E). GO analysis of these overlapping DEGs revealed an enrichment of GO terms associated with cardiac development majorly in downregulated genes in DS/CHD cells, and an enrichment of GO terms associated with nervous development in upregulated genes in DS/CHD cells. This may indicate that T21 might affect cell adhesion, cell communication, and cell fate specification during heart development (Figures 1E and 2F). Importantly, multiple cardiac gene sets altered in DS/CHD differentiating cells were significantly rescued by JAKi treatment (Figures 2F and S6C), indicating that inhibition of IFN signaling effectively reversed abnormalities in cardiac differentiation of DS/CHD iPSCs *in vitro*.

Video S3. Spontaneous contraction of clusters in cultures differentiated from control iPSCs (C68) treated with DMSO on day 13 post-induction of differentiation, related to Figure 2

Video S4. Spontaneous contraction of clusters in cultures differentiated from DS/CHD iPSCs (D49) treated with DMSO on day 13 post-induction of differentiation, related to Figure 2

Video S5. Spontaneous contraction of clusters in cultures differentiated from DS/CHD iPSCs (D49) treated with 1 μM JAKi on day 13 post-induction of differentiation, related to Figure 2

### IFN hyperactivity impairs early cardiac differentiation via downregulation of Wnt signaling

**Figure 3:**
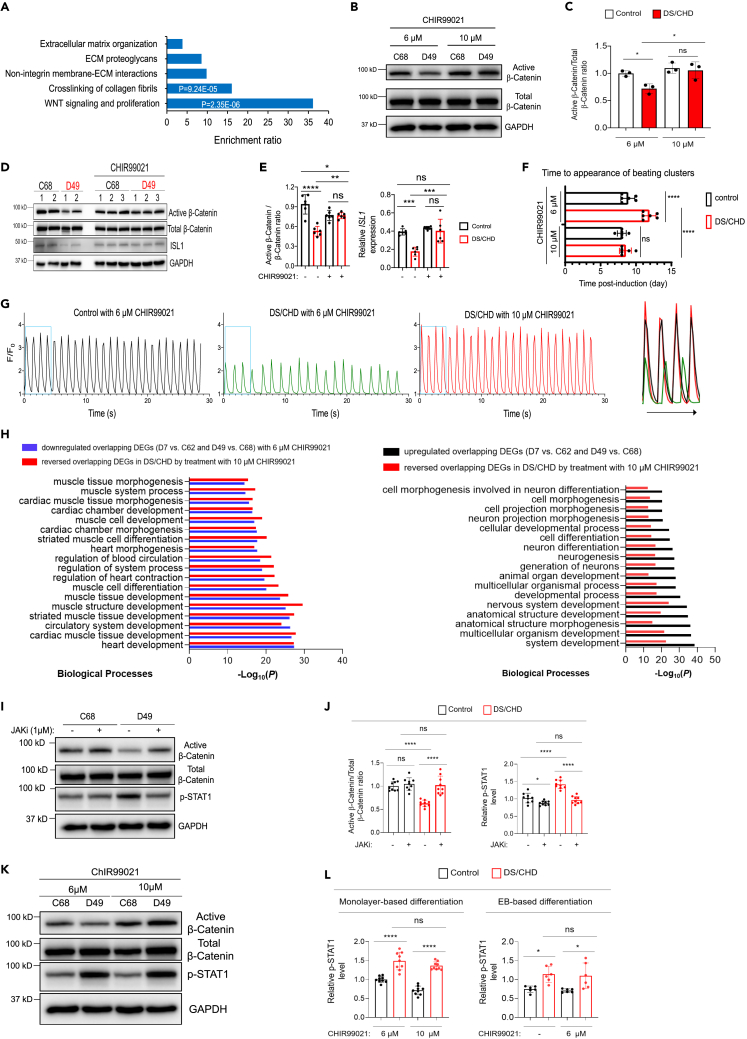
Restoration of the Wnt/β-Catenin pathway normalizes cardiac differentiation of DS/CHD iPSCs (A) REACTOME analysis of downregulated genes in DS/CHD cells on differentiation day 3. On day 3 post-induction of differentiation, RNA-seq was performed on C62, C68, D7, and D49 iPSC lines. Genes with expression ≥25 FPKM that were downregulated ≥1.5-fold (p < 0.05) were selected for REACTOME analysis. (B) Representative immunoblotting for indicated proteins in control and DS/CHD cells on differentiation day 3. Cells were cultured with either 6 or 10 μM CHIR99021, an activator of the Wnt/β-Catenin pathway between day 0 and day 1. (C) Quantification of the active β-Catenin/total β-Catenin ratio by immunoblotting. Each filled circle represents an individual iPSC line of 3 control (C42, C62, and C68) and 3 DS/CHD (D19, D7, and D49). Data are presented as mean ± SD. ∗p < 0.05, ns, not significant, ordinary one-way ANOVA. (D and E) Immunoblotting for indicated proteins in EBs on day 4. Differentiation of control (C68 and C42) or DS/CHD (D49 and D19) iPSCs was induced by the EB-based differentiation protocol, with or without 6 μM ChIR99021 from day 2 to day 4. A representative immunoblot is shown in D. Quantification of the active β-Catenin/total β-Catenin ratio and *ISL1* expression is shown in E. Data are presented as mean ± SD. Each filled circle represents one independent experiment, with 3 experiments for each iPSC line. ∗p < 0.05, ∗∗p < 0.01, ∗∗∗p < 0.001, ∗∗∗∗p < 0.0001, ns, not significant, Ordinary one-way ANOVA. (F) Time to the appearance of beating clusters in control or DS/CHD cultures post-induction of monolayer-based differentiation. Controls (C62 and C68) and DS/CHD (D7 and D49) cultures were treated with 6 or 10 μM CHIR99021 between day 0 and day 1. Each filled circle represents one independent experiment, with 3 experiments for each iPSC line. Data are presented as mean ± SD. ∗∗∗∗p < 0.0001, ns, not significant, ordinary one-way ANOVA. (G) Representative traces of Ca^2+^ transients measured with the Ca^2+^ indicator GCaMP6f in control iPSC-CMs (C68), DS/CHD iPSC-CMs (D49) with 6 μM CHIR99021, and DS/CHD iPSC-CMs with 10 μM CHIR99021. Control and DS/CHD iPSCs were induced to differentiation using the monolayer protocol. Ca^2+^ imaging was acquired on day 13. Merged traces in the box are shown on right. Quantification of Ca^2+^ transient amplitude and frequency is shown in Figure S3. (H) GO analysis of the overlapping down- and upregulated DEGs in DS/CHD-differentiated cells on day 7 and the rescue effects of restoration of the activity of the Wnt signaling. Three independent experiments were performed for each iPSC line (C62, C68, D7, and D49) under each condition for RNA-seq. FPKM ≥30 was set as cutoff to filter transcripts. Fold change ≥1.5 and p < 0.05 were set as criteria to determine DEGs. (I and J) Immunoblotting for indicated proteins in cells differentiated from 3 control (C62, C68, and C42) and 3 DS/CHD (D7, D49, and D19) iPSC lines on day 3. Cells were treated with vehicle or 1 μM JAKi from day 0 to day 3. Representative immunoblots are shown in I. Protein quantification is shown in J. Data are presented as mean ± SD. Three independent experiments were performed for each iPSC line. Each filled circle represents one independent experiment for an individual iPSC line. ∗∗∗∗p < 0.0001, ns, not significant, ordinary one-way ANOVA. (K and L) Immunoblotting for indicated proteins in cells differentiated from 3 control (C62, C68, and C42) and 3 DS/CHD (D7, D49, and D19) iPSC lines on day 3 using the monolayer-based differentiation protocol or in cells differentiated from 2 control (C68 and C42) and 2 DS/CHD (D49 and D19) iPSC lines on day 4 using the EB-based differentiation protocol. Monolayer-based differentiating cells were cultured with either 6 or 10 μM CHIR99021 from day 0 to day 1. EBs were treated with vehicle or 6 μM ChIR99021 from day 2 to day 4. Representative immunoblots are shown in K. Protein quantification is shown in L. Data are presented as mean ± SD. Three independent experiments were performed for each iPSC line. Each filled circle represents one independent experiment for an individual iPSC line. ∗p < 0.05, ∗∗∗∗p < 0.0001, ns, not significant, Ordinary one-way ANOVA.

Downregulated genes in DS/CHD cells on differentiation day 7 are significantly associated with cardiac development terms (Figure 1C). To further interrogate the mechanisms responsible for abnormal cardiac development in DS, we investigated signaling pathways that are dysregulated in cardiac differentiation of DS/CHD iPSCs, using REACTOME analysis of genes significantly downregulated by > 1.5-fold in DS/CHD cells on differentiation day 3. This analysis demonstrated that genes related to Wnt signaling were profoundly downregulated in DS/CHD cultures (Figure 3A). Activation of canonical Wnt/β-catenin signaling (hereafter referred to as Wnt signaling) is essential for early cardiogenesis.^29^^,^^30^^,^^31^^,^^32^^,^^33^^,^^34^^,^^35^^,^^36^^,^^37^ Therefore, we examined Wnt signaling by analyzing β-catenin activation through dephosphorylation at Ser37/Thr41. Active β-catenin was substantially reduced in DS/CHD iPSC-derived cells on differentiation day 3 (Figures 3B and 3C), indicating reduced Wnt signaling. Next, we examined whether increasing Wnt signaling activity could rescue the phenotype of cardiac differentiation of DS/CHD iPSCs. CHIR99021, a GSK-3 inhibitor, has been used to induce Wnt signaling in monolayer-based cardiac differentiation of human iPSCs.^45^^,^^46^^,^^47^ Our standard differentiation protocol uses 6 μM of CHIR99021 from day 0 to day 1 for monolayer-based iPSC differentiation. Treatment with 10 μM of CHIR99021 from day 0 to day 1 did not significantly alter the ratio of active β-catenin/total β-catenin in control cells, but significantly increased active β-catenin levels as well as the ratio of active β-catenin/total β-catenin in DS/CHD cells (Figures 3B and 3C).

To exclude potential effects of CHIR99021 on the Wnt pathway in DS cells, we differentiated iPSCs without adding any Wnt manipulators utilizing an EB-based differentiation protocol wherein the activity of Wnt signaling reached the peak at day 4.^55^ Immunoblotting revealed that Wnt signaling activity and expression of CPC marker *ISL1* decreased in DS/CHD cells at day 4 compared to control cells (Figures 3D and 3E). CHIR99021 treatment increased the activity of the canonical Wnt pathway and expression of cardiac gene *ISL1* in DS/CHD cells (Figures 3D and 3E). Moreover, monolayer-based cardiac differentiation of DS/CHD iPSCs in the presence of 10 μM of CHIR99021 is comparable to control iPSCs, as shown by appearance time of beating clusters (Figure 3F), amplitude and frequency of Ca^2+^ transients (Figures 3G, S3A, and S3B), and contractile frequency and strength (Videos S6 and S7). Interestingly, treatment with 10 μM CHIR99021 did not dramatically increase the activity of Wnt signaling and cardiogenesis in control iPSCs (Figures 3B–3F). Taken together, these studies using both monolayer-based differentiation system and EB-based differentiation system demonstrate that normalization of the Wnt pathway, which is significantly downregulated during early differentiation of DS/CHD iPSCs, reverses defects in cardiac differentiation.

Video S6. Spontaneous contraction of clusters in cultures differentiated from DS/CHD iPSCs (D7) treated with 6 μM of CHIR99021 on day 13 post-induction of differentiation, related to Figure 3

Video S7. Spontaneous contraction of clusters in cultures differentiated from DS/CHD iPSCs (D7) treated with 10 μM of CHIR99021 on day 13 post-induction of differentiation, related to Figure 3

To further investigate molecular mechanisms by which activation of Wnt signaling rescued cardiac differentiation of DS/CHD iPSCs, we performed RNA-seq analysis to profile global gene expression in cells differentiated from control and DS/CHD iPSCs (C62 and C68 versus D7 and D49) with lower or higher activity of canonical Wnt signaling. We performed transcriptomic analysis on differentiation day 7. The expression of 305 of 501 upregulated DEGs and 344 of 444 downregulated DEGs between control and DS/CHD was effectively reversed by increased Wnt activity. GO analysis of DEGs revealed that an enrichment of downregulated genes in DS/CHD cells that were reversed was particularly associated with cardiac development terms, indicating that cardiac differentiation of DS/CHD iPSCs is rescued by restoration of the Wnt pathway (Figures 3H and S7). These data indicate that reduced Wnt signaling contributes to defects in cardiac differentiation in DS *in vitro*.

Next, we investigated the interplay between IFN hyperactivity and reduced Wnt signaling during cardiogenesis in DS/CHD iPSCs. IFNα treatment of control cells significantly increased the activity of IFN signaling and dramatically decreased the activity of Wnt signaling during the differentiation of iPSCs (Figures S8A–S8D). Hyperactivated IFN signaling in control cells significantly decreased the frequency and strength of Ca^2+^ transients of beating clusters to the level of DS/CHD (Figures S3A–S3B, and S8E), indicating the detrimental effect of IFN hyperactivity on cardiac differentiation of iPSCs. These data indicate that IFN hyperactivity is sufficient to cause T21 phenotypes. Strikingly, the normalization of IFN signaling by JAKi not only ameliorated cardiac differentiation of DS/CHD iPSCs but also restored Wnt signaling (Figures 3I and 3J). In contrast, normalization of the Wnt signaling pathway ameliorated cardiac differentiation of DS/CHD iPSCs (Figures 3B–3H) but did not significantly affect IFN signaling (Figures 3K and 3L). In other words, hyperactive IFN signaling did not impair cardiac differentiation of DS/CHD iPSCs once the Wnt pathway was normalized in these cells.

Altogether, these data indicate that IFN hyperactivity impairs *in vitro* cardiogenesis in DS by downregulation of Wnt signaling.

### JAK inhibition restores Wnt signaling and prevents cardiogenic dysregulation in Dp16 mice

**Figure 4:**
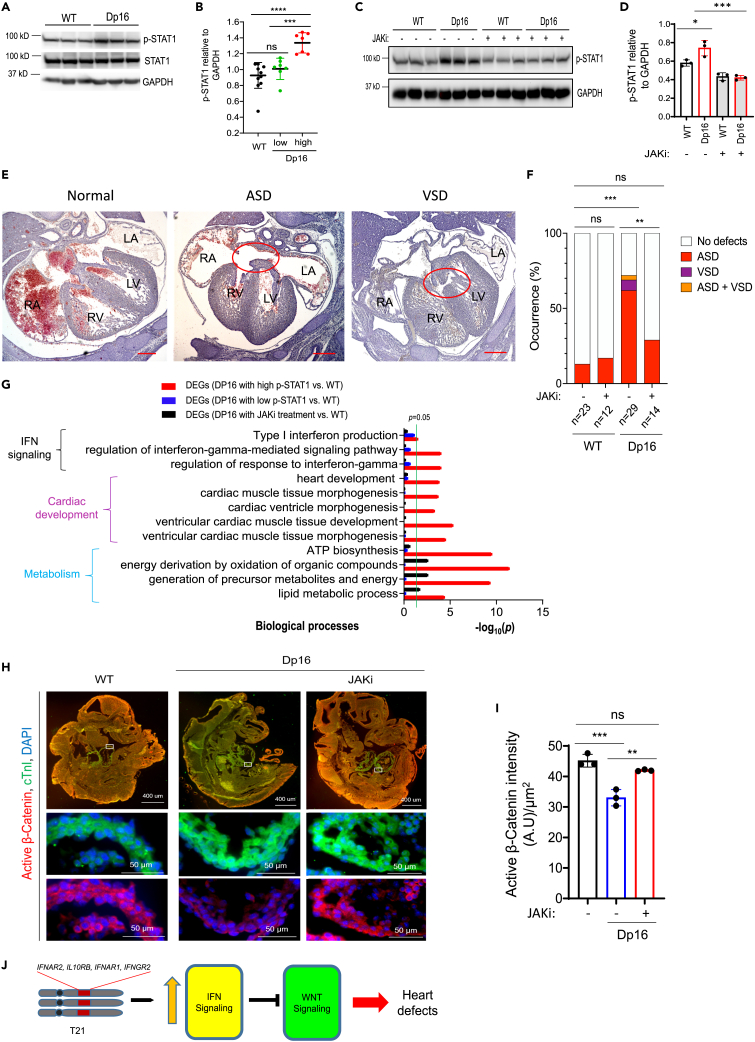
Pharmacological inhibition of IFN signaling prevents heart malformations in Dp16 embryos (A and B) Immunoblotting analysis of heart extracts from indicated embryos at E15.5 for p-STAT1, total STAT1, and GAPDH. Representative western blots are shown in A. Quantification of protein levels from western blots are in B. Each filled circle represents one individual embryonic heart. Data are presented as mean ± SD. ∗∗∗p < 0.001, ∗∗∗∗p < 0.0001, ordinary one-way ANOVA. (C and D) Immunoblotting analysis of heart extracts from indicated embryos at E15.5 for p-STAT1 and GAPDH. Pregnant mice were daily treated with the JAKi (10 mg/kg body weight/day, i.p. injection) beginning on day 6.5 post-conception. Hearts were harvested at E15.5 for analysis. Protein quantification is shown in D. Each filled circle represents one individual heart. Data are presented as mean ± SD. ∗p < 0.05, ∗∗∗p < 0.001, Ordinary one-way ANOVA. (E and F) Histological analysis of cardiac septation in embryos at E15.5. Representative images of hematoxylin & eosin-stained sections of embryos are shown in E. Normal septation of four chambers in a WT embryo, and atrial septal defect (ASD) or ventricular septal defect (VSD), which were each observed in Dp16 embryos. LA, left atrium; RA, right atrium; LV, left ventricle; RV, right ventricle. Percentages of E15.5 embryos displaying heart malformations are shown in F. Scale bar, 300 μm. ∗∗p < 0.01, ∗∗∗p < 0.001, ns, not significant, Fisher’s exact test. (G) GO analysis of the dysregulated DEGs, as assessed by RNA-seq, associated with variable IFN signaling and JAKi treatment. FPKM ≥30 in WT samples was set as cutoff to filter transcripts. DEGs were determined by Student’s *t* test (p < 0.05). N = 3–4 hearts for each group. (H and I) Examination of cardiac troponin I (cTnI) and active β-Catenin levels in embryos at E9.5 by immunofluorescence (IF) analysis. Pregnant mice were treated daily with vehicle or the JAKi (10 mg/kg body weight/day, i.p.) beginning with day 6.5 post-conception. Embryos were harvested at E9.5 for IF analysis. Representative IF images are presented in H. Scale bars on top panels, 400 μm. Scale bars on middle and bottom panels, 50 μm. Each white box indicates area of magnification in bottom images. Quantification of IF signaling intensity for active β-Catenin in cTnI^+^ developing hearts in I. Two sections were analyzed for each embryo. Each filled circle represents the average of active β-Catenin signals in one embryo. ∗∗p < 0.05, ∗∗∗p < 0.05, ns, not significant, ordinary one-way ANOVA. (J) T21 leads to increased expression of IFN receptors encoded by genes on HSA21, which overactivates IFN signaling. Activated IFN signaling decreases the activity of the canonical Wnt pathway, eventually leading to defects during heart development.

Next, we examined the potential of JAKi to normalize cardiogenesis in DS *in vivo* using the Dp16 mouse model. CHDs are observed in ∼50% of people with DS, as well as ∼50% of Dp16 mice.^17^ We hypothesized that the activity of IFN signaling could be highly variable in Dp16 embryonic hearts, potentially contributing to CHD occurrence. We performed immunoblotting for p-STAT1 in developing hearts from wild type (WT) and Dp16 at E15.5. Interestingly, p-STAT1 levels were higher but variable in Dp16 versus WT (Figures 4A and 4B), which could potentially explain the variable penetrance of CHDs in Dp16 mice. Next, we sought to determine whether normalization of the IFN pathways by JAKi could prevent heart defects in Dp16 mice. We crossed Dp16 male mice to WT females. Pregnant females were treated with JAKi daily at a dose of 10 mg/kg/day beginning on 6.5 days after conception (Figure S9). This JAKi dosage was determined based on previous studies,^56^^,^^57^ and normalized levels of p-STAT1 in Dp16 hearts at E15.5 (Figures 4C and 4D).

The mouse heart achieves its prenatal morphogenesis with the development of 4-chamber and septation by E15.5.^23^^,^^58^ Therefore, we examined heart morphology at E15.5 by H&E staining of serial sections (Figure S10). We observed heart defects including ASD, VSD, and ASD/VSD, in 72% of Dp16 embryos, compared to only 15% of WT embryos at E15.5 (Figures 4E and 4F), similar to a previous report wherein the occurrence of heart defects in Dp1Tyb with trisomy of chromosome 16 and WT mice at E14.5 is 62% and 27%, respectively.^59^ Strikingly, JAKi treatment significantly decreased the occurrence of heart abnormalities in Dp16 mice at E15.5 from 72% to 29%, without affecting WT embryos (Figure 4F), indicating that pharmacological attenuation of IFN signaling prevents abnormalities during heart development in Dp16 mice. To investigate the underlying mechanisms by which JAKi rescues defects in Dp16 heart development, we profiled global gene expression in WT and Dp16 hearts at E15.5 treated with or without JAKi using RNA-seq. DEGs between Dp16 hearts with high p-STAT1 levels but not Dp16 hearts with low p-STAT1 levels as shown in Figure 4B and WT hearts are significantly associated with terms of IFN signaling, cardiac development, and metabolism (Figure 4G), suggesting a crucial role of IFN signaling hyperactivation in the development of heart defects in Dp16 mice. These terms altered in the Dp16 heart were dramatically rescued by JAKi treatment (Figure 4G), indicating that normalization of IFN signaling effectively reversed abnormal gene expression programs associated with heart malformations in DS *in vivo*.

The Wnt pathway is activated during early heart development in mice (e.g. E8.5–E10.5).^34^^,^^37^ Immunofluorescence assays demonstrated that the activity of Wnt signaling was significantly downregulated in Dp16 mice at E9.5 (Figure 4H). These *in vivo* data are consistent with our previous findings that the Wnt pathway is downregulated during early cardiac differentiation of DS/CHD iPSCs (Figures 3A–3H). Treatment with JAKi, which prevented abnormal heart development in Dp16 mice (Figures 4E–4G), also normalized the activity of Wnt signaling in Dp16 developing hearts at E9.5 (Figures 4H and 4I).

Therefore, our *in vitro* and *in vivo* data strongly support that IFN hyperactivity impairs cardiogenesis in DS by dysregulating the Wnt pathway (Figure 4J).

## Discussion

CHDs are very frequent in children with DS, with a prevalence of ∼50% compared to less than 1% in typical children. The mechanisms by which T21 causes cardiogenic dysregulation remain elusive. Using a combination of the human iPSC-based model and a mouse model for DS, Dp16, we illuminated a pathway involving increased dosage of IFNRs encoded on chromosome 21 and downstream downregulation of Wnt signaling as a driver of defects in cardiogenesis in DS. Triplication of chromosome 21 causes IFNR overexpression concurrent with hyperactive IFN signaling and downregulated Wnt signaling during cardiogenesis in DS. Normalization of IFN signaling restored Wnt signaling and rescued defects in cardiogenesis in DS *in vitro* and *in vivo*. Normalization of the Wnt pathway rescued cardiogenesis, while not affecting the activity of IFN pathways. Therefore, our studies demonstrate that hyperactivation of IFN signaling causes abnormal cardiogenesis in DS by dysregulation of the Wnt pathway (Figure 4J).

CHDs are the most common developmental abnormality in newborns, with a prevalence of ∼0.9%.^60^ It is estimated that more than 2 million people are living with CHDs in the USA.^61^ Increased levels of inflammatory cytokines have been observed in children with CHDs,^62^^,^^63^ indicating that dysregulation of inflammatory pathways may impact fetal heart development. Analysis of genomic sequences in several hundred individuals with DS identified a few genetic variants in the IFNR genes located on HSA21.^20^^,^^21^ These studies indicate crucial roles of inflammatory pathways in the risk of CHDs. However, the underlying mechanisms are not well known. We found that hyperactivation of IFN signaling is a causative factor of impaired cardiac differentiation of DS/CHD iPSCs (Figures 2 and S5) and cardiogenic dysregulation in Dp16 mice (Figure 4). Furthermore, treatment of pregnant mice with JAKi improved heart development in Dp16 embryos (Figures 4E–4G). Our findings that treatment with a JAKi rescued defects in human iPSC cardiogenesis (Figure 2) and Dp16 mouse heart development (Figures 4E–4G) serve as a proof of concept that pharmacological intervention could treat heart malformations in DS. The JAKi used in this study is able to suppress all three types of IFN signaling, as they all employ JAK1 for signal transduction. IFN-mediated immune responses are central to host defense against infection^64^ and attenuating type I IFN pathway may increase the risk of maternal infections during pregnancy.^65^ Therefore, it is necessary to conduct further studies focusing on distinguishing targets of IFN signaling that are drivers of heart defects from those that are essential for host defense against microbial infection. Building upon these studies, we would be able to selectively suppress detrimental aspects of IFN signaling in DS.

CHD survivors are at greater risk of cardiovascular disease such as cardiac arrhythmia and heart failure,^23^^,^^66^^,^^67^ but the underlying mechanisms remain elusive. Circulating pro-inflammatory cytokines are significantly increased in patients with heart failure,^68^^,^^69^^,^^70^ as well as in people with DS.^51^ IFN signaling pathways play distinctive roles in cardiovascular disease. Knockout of *Ifng* exaggerated cardiac hypertrophy in systolic and diastolic heart failure,^71^^,^^72^ indicating a protective role of the IFNγ-mediated type II IFN signaling in cardiac pathogenesis. However, treatment with an IFNAR-neutralizing antibody improved cardiac function and survival post-myocardial infarction,^73^ suggesting a deleterious effect of the type I IFN pathway in response to myocardial infarction. Knockdown of *IFNAR1/2* attenuated defects in cardiac differentiation of DS/CHD iPSCs (Figure S5). Further studies focusing on determining the association of individual IFN pathways with the risk of CHDs and cardiomyopathy/heart failure in people with DS could identify targets for future therapy.

The Wnt signaling pathway is highly conserved among species. Temporal regulation Wnt signaling is required for cardiac development. Activation of the Wnt pathway is required for the expansion of CPCs during early development stages.^29^^,^^30^^,^^31^^,^^32^^,^^34^ Decreased activity of the canonical Wnt pathway occurred during early differentiation of DS/CHD iPSCs (Figures 3A–3E) and early heart development in Dp16 embryos (Figures 4H and 4I). Treatment with CHIR99021, a GSK-3 inhibitor that enhances the activity of canonical Wnt signaling, improved cardiac differentiation of DS/CHD iPSCs (Figures 3B–3H and Videos S6 and S7). Highly potent and selective GSK-3 inhibitors have been investigated to treat neurodegenerative disorders in preclinical and clinical studies.^74^ The Wnt pathway is essential for cardiovascular, neuronal, craniofacial, and musculoskeletal development. The development of cardiovascular, neurological, and musculoskeletal systems is particularly affected in individuals with DS.^1^^,^^2^^,^^3^ The Wnt pathway was dramatically downregulated in the entire Dp16 embryo at E9.5, restored by the JAKi treatment (Figure 4H), indicating that the downregulation of Wnt signaling could contribute to DS hallmarks, across multiple organ systems. Therefore, our findings hold the potential for multidimensional therapeutic endpoints in DS by modulation of Wnt signaling.

Crosstalk between the Wnt pathway and IFN signaling has been shown in previous studies.^75^^,^^76^ For example, the Wnt pathway can play a role as either a positive or negative regulator of the type I IFN signaling pathway in response to viral infection. Treatment of pregnant mice with JAKi increased the activity of Wnt signaling (Figures 4H and 4I) and prevented heart defects in Dp16 embryos (Figures 4E–4G). However, increasing the activity of Wnt signaling using a GSK3 inhibitor did not affect the activity of IFN signaling (Figures 3K and 3L). These findings reveal that hyperactivation of IFN signaling causes defects in cardiogenesis in DS by downregulating the activity of the Wnt pathway. Recently, Lana-Elola et al.^59^ identified a 4.9 Mb genome region (from *Mir802* to *Zbtb21*) that when present in 3 copies, was sufficient to cause cardiac abnormalities. This 4.9 Mb genomic region overlapping with the previously identified 3.7 Mb region^12^ contains the *Dyrk1a* gene, which functions as an inhibitor of Wnt signaling in some contexts.^77^ Future studies focusing on defining mechanisms by which IFN signaling hyperactivation downregulates Wnt signaling would greatly aid in discovering effective therapies to treat DS.

### Limitations of the study

Using human iPSCs derived from healthy donors and individuals with DS and CHDs, we reveal that hyperactivation of the IFN pathways causes cardiogenic defects in DS by downregulating the Wnt signaling pathway. Studies using the Dp16 mouse model also demonstrate that IFN hyperactivity downregulates the Wnt pathway and disrupts cardiogenesis in DS *in vivo*. The iPSC platform provides a tool to study molecular and cellular mechanisms to control human cardiogenesis. However, the monolayer-based (Figure S2) or EB-based (Figures 3D, 3E, and 3L) cardiac differentiation of iPSCs cannot completely mimic human heart development *in vivo*. More powerful and faithful models like the human heart organoid platform would better understand heart defects in people with DS.

Our current study focuses on signaling pathways that dysregulate cardiogenesis in DS. It is likely that defects in cardiogenesis eventually lead to CHDs at birth. However, this study cannot directly address these questions: (1) whether IFN hyperactivity causes CHDs by downregulating the Wnt pathway, (2) how IFN hyperactivity causes many types of CHDs in DS,^1^^,^^2^^,^^3^ and (3) whether JAKi can be used to prevent CHDs in DS. Our future studies on examining heart defects in multiple mouse models of DS (e.g., Ts65Dn,^78^ Tc1,^79^ and Dp16) around and after birth will be performed to address these questions.

## STAR★Methods

### Key resources table

**Table:** 

REAGENT or RESOURCE	SOURCE	IDENTIFIER
**Antibodies**		

Rabbit polyclonal anti-Nanog	Cell Signaling Technology	Cat#3580S; RRID:AB_2150399
Mouse monoclonal anti-SSEA-4	Developmental Studies Hybridoma Bank	Cat#MC-813-70; RRID:AB_528477
Mouse monoclonal anti-TRA-2-49	Developmental Studies Hybridoma Bank	Cat#TRA-2-49/6E; RRID:AB_528073
Rabbit polyclonal anti-cTnI	PhosphoSolutions	Cat#2010-TNI; RRID:AB_2492267
Mouse monoclonal anti-active β-Catenin	Millipore	Cat#05-665; RRID:AB_309887
Rabbit polyclonal anti-total β-Catenin	Cell signaling	Cat#9581S; RRID:AB_490891
Mouse monoclonal anti-ISL1	Developmental Studies Hybridoma Bank	Cat#40.2D6; RRID:AB_528315
Rabbit monoclonal anti-Phospho-STAT1 (Tyr701)	Cell signaling	Cat#9167S; RRID:AB_561284
Rabbit polyclonal anti-total STAT1	Cell signaling	Cat#9172S; RRID:AB_2198300
Mouse monoclonal anti-GATA4	Santa Cruz	Cat#sc-25310; RRID:AB_627667
Mouse monoclonal anti-GAPDH	Ambion	Cat#AM4300; RRID:AB_437392

**Bacterial and virus strains**		

Lentivirus carrying shRNAs against scrambled target	Functional Genomics Facility of University of Colorado Cancer Center Shared Resource; https://functionalgenomicsfacility.org/product/	SHC002
Lentivirus carrying shRNAs against human IFNAR1	Functional Genomics Facility of University of Colorado Cancer Center Shared Resource; https://functionalgenomicsfacility.org/product/	TRCN0000059013
Lentivirus carrying shRNAs against human IFNAR2	Functional Genomics Facility of University of Colorado Cancer Center Shared Resource; https://functionalgenomicsfacility.org/product/	TRCN0000058786
Adenovirus carrying CAG-GCaMP6f	SignaGen Laboratories	Cat#SL101144

**Chemicals, peptides, and recombinant proteins**		

MatriGel	Corning	Cat#354277
Gentle Cell Dissociation Reagent	STEMCELL Technologies	Cat#07174
mTeSR1	STEMCELL Technologies	Cat#85850
Y-27632	Enzo Life Sciences	Cat#ALX-270-333-M025
Accumax	STEMCELL Technologies	Cat#07921
RPMI1640	Life Technologies	Cat#11875093
B27 supplement, minus insulin	Life Technologies	Cat#A1895601
B27 supplement	Life Technologies	Cat#17504044
ChIR99021	Cayman	Cat#252917-06-9
IWP2	Thermo Fisher Scientifics	Cat#35-331-0
Puromycin	Thermo Fisher Scientific	Cat#J67236.8EQ
VECTASHIELD mounting medium with DAPI	Vector Laboratories	Cat#H-1200
Hoechst 33342	Molecular Probes	Cat#H3570
cOmplete, Mini Protease Inhibitor Cocktail	Roche	Cat#11836153001
Halt Phosphatase Inhibitor Cocktail	Thermo Scientific	Cat#78420
TRIzol	Invitrogen	Cat#15596026
TURBO DNase	Invitrogen	Cat#AM2238
iScript Reverse Transcription Supermix	Bio-rad	Cat#1708840
SYBR Green PCR Master Mix	Applied Biosystems	Cat#4309155
Tofacitinib	LC Laboratories	Cat#T-1399
IFN-alpha A	R&D Systems	Cat#111012
Paraformaldehyde	Thermo Fisher Scientific	Cat#J19943.K2
DMEM/F12	Thermo Fisher Scientific	Cat#10565018
Knockout Serum Replacement	Thermo Fisher Scientific	Cat#10828028
MEM nonessential amino acids	Thermo Fisher Scientific	Cat#11140050
GlutaMAX	Thermo Fisher Scientific	Cat#35050-061
β-mercaptoethanol	Thermo Fisher Scientific	Cat#31350010
Fetal Bovine Serum (FBS)	Thermo Fisher Scientific	Cat#26140079
Bovine Serum Albumin (BSA)	Sigma-aldrich	Cat#A3294
Goat serum	Sigma-aldrich	Cat# G9023

**Critical commercial assays**		

KaryoStat+ Assay Service	Thermo Fisher Scientific	Cat#A52849

**Deposited data**		

Raw and analyzed RNA-seq data	This paper	GEO: GSE217557

**Experimental models: Cell lines**		

Human iPSC lines derived from healthy controls, see Table S1	Crnic Institute Human Trisome Project (HTP)	N/A
Human iPSC lines derived from DS/CHD patients, see Table S1	Crnic Institute Human Trisome Project (HTP)	N/A

**Experimental models: Organisms/strains**		

Mouse: C57BL/6J	The Jackson Laboratory	RRID:IMSR_JAX:000664
Mouse: Dp(16Lipi-Zbtb21)1Yey/J	The Jackson Laboratory	RRID:IMSR_JAX013530

**Oligonucleotides**		

qPCR primers, see Table S3	This paper	N/A

**Software and algorithms**		

STAR aligner v2.7	http://code.google.com/p/rna-star/	RRID:SCR_004463
RsubRead	https://bioconductor.org/packages/release/bioc/html/Rsubread.html	RRID:SCR_016945
DESeq2	https://bioconductor.org/packages/release/bioc/html/DESeq2.html	RRID:SCR_015687
Gene Set Enrichment Analysis (GSEA)	https://www.gsea-msigdb.org/gsea/index.jsp	RRID:SCR_003199
PANTHER	http://www.pantherdb.org/	RRID:SCR_004869
ImageJ	https://imagej.nih.gov/ij/	RRID: SCR_003070
GraphPad Prism	GraphPad Software, LLC	RRID:SCR_002798

### Resource availability

#### Lead contact

Further information and requests for resources and reagents should be directed to and will be fulfilled by the lead contact, Kunhua Song (kunhua.song@cuanschutz.edu).

#### Materials availability

This study did not generate new unique reagents.

### Experimental model and study participant details

All animal research complied with the protocols (#00319) approved by the Institutional Animal Care and Use Committee (IACUC) of the University of Colorado Anschutz Medical Campus. Dp(16Lipi-Zbtb21)1Yey/J (hereafter “Dp16”) mice, a mouse model of DS/CHD, were purchased from the Jackson Laboratory (Cat# JAX:013530, RRID:IMSR_JAX013530). Both male and female mice at E15.5 were randomly assigned to experimental groups. The phenotype described in the study is gender independent.

Human iPSC lines were obtained from the Crnic Institute Human Trisome Project (HTP), under a study protocol approved by the Colorado Multiple Institutional Review Board (COMIRB 15-2170, NCT02864108, see also www.trisome.org). Detailed information, including gender and age on human iPSC lines in this study is presented in Table S1.

### Method details

#### Culture and characterization of human iPSCs

Human iPSCs (hiPSCs) were cultured in mTeSR1 in a 12-well plate coated with MatriGel (Corning). mTeSR1 was refreshed daily. The pluripotency of the hiPSCs was examined by examination of the expression of pluripotency markers Nanog, SSEA-4 and TRA-2-49. Karyotyping was performed by *WiCell* Research Institute and Molecular Pathology Shared Resource Cytogenetics at the University of Colorado for G-banding assay and Thermo Fisher Scientific for KaryoStat+ ^TM^ Assay.

#### Monolayer cardiac differentiation of hiPSCs into CMs

hiPSCs were dissociated with Accumax (STEMCELL Technologies) and replated in a 24-well plate pre-coated with MatriGel. Cells were incubated in 1 mL of mTeSR1 per well for 4 days before induction. GSK3 Inhibitor CHIR99021 (Cayman) was applied at induction day 0 to day 1 in RPMI1640 (Life Technologies) with B27 supplement minus insulin (Life Technologies). Cells were incubated with IWP2 (Thermo Fisher Scientific) from day 3 to day 5. On day 5, cells were incubated in fresh RPMI1640 with B27 supplement minus insulin. Cells were then cultured in RPMI1640 with B27 (Life Technologies). For JAKi treatment, 1 μM Tofacitinib (LC Laboratories) was added to the culture medium from differentiation day 0 to day 3. For IFNα treatment, 10 ng/mL IFNα (R&D Systems) was added to the culture medium from differentiation day 0 to the end of the experiment.

#### Embryoid body differentiation of hiPSCs

Embryoid bodies (EBs) were generated from hiPSCs by adapting the methods reported in a previous study^55^ with modifications. Briefly, hiPSCs were seeded on AggreWell 800 Plates (STEMCELL) with UM/F- medium supplied with 10 μM Y-27632 (Enzo Life Sciences). UM/F- medium composed of DMEM/F12 (Thermo Fisher Scientific) containing 20% Knockout Serum Replacement (Thermo Fisher Scientific), 1× MEM nonessential amino acids (Thermo Fisher Scientific), 1x GlutaMAX (Thermo Fisher Scientific) and 0.1 mM β-mercaptoethanol (Thermo Fisher Scientific). hiPSCs were cultured with UM/F- medium supplied with 10 μM Y-27632 for 5 days (medium refreshed every two days) to allow the formation of spheroids. On day 6, EBs were resuspended in fresh UM/F- medium supplied with 10 μM Y-27632 and transferred to regular cell culture plates. On the next day (differentiation day 1), EBs were cultured with EB20 medium composed of DMEM/F12 containing 20% FBS (Thermo Fisher Scientific), 1× MEM nonessential amino acids, 1x GlutaMAX and 0.1 mM β-mercaptoethanol. For CHIR99021 treatment, 6 μM CHIR99021 was supplemented to EB20 medium from differentiation day 2 to day 4.

#### Lentivirus-mediated gene knockdown in hiPSCs

Lentivirus particles carrying shRNAs against scrambled target (SHC002), IFNAR1 (TRCN0000059013), or IFNAR2 (TRCN0000058786) were purchased from the Functional Genomics Facility of the University of Colorado Anschutz. Stable hiPSC clones were selected after lentiviral infection (MOI≈1) by culturing with mTeSR1 medium supplied with 1μg/mL puromycin (Thermo Fisher Scientific) for one week. These hiPSCs were then expanded for experiments.

#### Transcriptomic analysis

*RNA preparation:* Total RNA from cells or mouse tissues was isolated using TRIzol reagent (Invitrogen) according to the manufacturer’s instructions. All RNA samples were treated with TURBO DNase (Invitrogen) to remove any genomic DNA contaminations before being used for experiments. RNA quality was examined using Tapestation (Agilent Technologies).

*Real-Time PCR:* cDNA was synthesized using the iScript Reverse Transcription Supermix (Biorad). qPCR was performed using SYBR PowerUP master mix (Applied Biosystems) on the StepOne Real-Time PCR System (Applied Biosystems). GAPDH was used as an internal control. qPCR primer sequences used in this study are listed in Table S3.

*RNA-seq:* The sequencing libraries preparation and RNA-seq were performed by BGI Genomics. Sequencing was performed on the DNBSEQ (DNBSEQ Technology) platform at a read depth of 40 million reads per sample using paired-end sequencing. FastQC was used for the raw RNA-seq reads for the control of sequence quality, GC content, the presence of adaptor sequences and duplicated reads and to ensure homogeneity of sequencing reads between samples. Reads that passed quality control were aligned to NCBI GRCh38 human reference genome or GRCm38/mm10 mouse reference genome using the splice-aware STAR aligner v2.7. The read counts were generated from the aligned reads with featureCounts function in the RsubRead package. The read counts were normalized with DESeq2 R package. The RNA-seq data generated in this study were deposited to the GEO Repository as GSE217557. We performed differential expression analysis using DESeq2 package where the normalized gene counts were modeled using negative binomial distribution and p-value adjustments for multiple testing, following Benjamini–Hochberg procedure. A 1.5-fold change threshold was used in conjunction with an adjusted 0.05 p-value threshold to identify differentially expressed genes. Gene set enrichment analysis was conducted using GSEA software (https://www.gsea-msigdb.org/gsea/index.jsp).^48^^,^^49^ Web-based gene ontology analysis was performed via PANTHER classification system (http://pantherdb.org).^80^

#### Histological analysis of mouse embryonic hearts

A male Dp16 mouse was crossed with a female WT C57BL/6J (The Jackson Laboratory). The female was checked daily for vaginal plugs. The female was separated from the male immediately in the morning (E0.5) of visual confirmation of vaginal plugs. The pregnant mice with E9.5 or E15.5 embryos were sacrificed by CO_2_ inhalation and cervical dislocation. For JAKi treatment, pregnant mice received Tofacitinib (LC Laboratories) (10 mg/kg body weight) daily via intraperitoneal injection (i.p.) starting from E6.5 to the day before embryo harvest. The yolk sac (for E9.5) or a tail snip (for E15.5) was collected for genotyping. Embryos were then fixed in 2% paraformaldehyde (PFA) at 4°C overnight. Fixed embryos were stored in 70% ethanol before being embedded in paraffin. Embedded embryos were sectioned sagittally (E9.5) or transversely (E15.5) at 7μm thickness using a LEICA RM 2155 Rotary Microtome. Serial sections were collected throughout the developing heart to assess heart malformations. Representative sections were chosen for Hematoxylin and Eosin (H&E) staining as previously described.^81^ Histological images were then captured using the Keyence BZ-X710 All-in-One Fluorescence Microscope.

#### Immunofluorescence staining and microscopy imaging

Cultured cells were fixed in 2% paraformaldehyde for 30 min at room temperature and then washed 3 times with 1x DPBS. The cells were permeabilized in 0.5% Triton X-100 for 20 min at room temperature. After blocking in 1x DPBS containing 10% horse serum for 30 min at room temperature, cells were incubated with primary antibodies in 10% horse serum in 1x DPBS for 1 hour at room temperature. Cells were washed 3 times with 1x DPBS and then incubated with secondary antibodies (Molecular Probes Alexa Fluor 555, 1:1,000) and Hoechst (Molecular Probes, 1:5,000) for 30 min at room temperature. Cells were washed 3 times with 1x DPBS before imaging by EVOS FL Cell Imaging System (Life Technologies). For paraffin-embedded mouse embryo sections, samples were first dewaxed and rehydrated by immersion in xylene for 9 mins, 100% ethanol for 9 mins, 95% ethanol for 3 mins, 70% ethanol for 3 mins, ddH_2_O for 5mins. Heat-induced epitope retrieval was performed in sodium citrate buffer (10 mM sodium citrate, 0.05% Tween 20, PH6.0) in a pressure cooker. Samples were treated with 0.3 M glycine (PH7.4) at room temperature for 30 mins to reduce autofluorescence before 30-min permeabilization in 0.25% Triton X-100. After washing 3 times in 1x DPBS and blocking with 1% BSA (Sigma) supplemented with 10% goat serum (Sigma), samples were incubated with primary antibodies at 4°C overnight. The next day, samples were washed 3 times with 1x DPBS and then incubated with secondary antibodies (Molecular Probes Alexa Fluor 488 or Alexa Fluor 555, 1:400) for 30 min at room temperature. Samples were washed 3 times with 1x DPBS before being mounted in VECTASHIELD mounting medium with DAPI (Vector Laboratories) for imaging. The primary antibodies include antibodies against Nanog (Cell Signaling Technology, 1:100), SSEA-4 (Developmental Studies Hybridoma Bank, 1:10), TRA- 2-49 (Developmental Studies Hybridoma Bank, 1:20), cTnI (PhosphoSolutions, 1:200), active β-Catenin (Millipore, 1:100). Stained samples were imaged using either an EVOS FL Cell Imaging System (Life Technologies) or a Keyence BZ-X710 All-in-One Fluorescence Microscope. The immunofluorescence intensities were quantified using ImageJ and graphed in GraphPad Prism 9.

#### Immunoblotting

Cultured cells were washed with ice-cold 1xDPBS twice before lysing in ice-cold lysis buffer (150 mM NaCl, 50 mM Tris-HCl pH 7.4, 1 mM EDTA, 1% Triton, with Complete mini tablet (Roche), 1 mM phenylmethylsulphonyl fluoride and 1x Halt Phosphatase Inhibitor Cocktail (Thermo Scientific) freshly added before use). Embryonic hearts were dissected from mouse embryos at E15.5 and froze immediately in liquid nitrogen. The tail from the same embryo was collected for genotyping. Frozen tissues were lysed and homogenized with SSB16 beads (Next Advance) in ice-cold lysis buffer (150 mM NaCl, 50 mM Tris-HCl pH 7.4, 1 mM EDTA, 1% Triton, with Complete mini tablet (Roche), 1 mM phenylmethylsulphonyl fluoride and 1x Halt Phosphatase Inhibitor Cocktail (Thermo Scientific) freshly added before use). 10 μg of total protein lysate per sample was loaded for Western blot analysis. The primary antibodies include anti-active β-Catenin (Millipore, 1:1000), anti-total β-Catenin (Cell signaling, 1:1000), anti-ISL1 (Developmental Studies Hybridoma Bank, 1:1000), anti-Phospho-STAT1 (Tyr701) (Cell signaling, 1:1000), anti-total STAT1 (Cell signaling, 1:1000), anti-GATA4 (Santa Cruz, 1:500), and anti-GAPDH (Ambion,1:5,000). The secondary antibodies include Goat Anti-Mouse IgG (Southern Biotech, 1:2,000) and Goat Anti-Rabbit IgG (Life Technologies, 1:2,000). The results were quantified by using ImageJ and graphed in GraphPad Prism 9.

#### Analysis of Ca^2+^ transients with GCaMP

hiPSC-CMs were infected with adenovirus carrying CAG-GCaMP6f^82^ (SignaGen Laboratories SL101144, MOI=10) at the day 10 post-cardiac induction. Ca^2+^ transients (fluorescence intensity) were captured (7-fps, 200 cycles) using a Zeiss LSM780 confocal microscope system at day 13 post-cardiac induction. Time-lapse imaging data were then exported to Microsoft Excel for analysis of Ca^2+^ transient amplitude and frequency. Fluorescence intensity was normalized to baseline (F/F_0_).

### Quantification and statistical analysis

All data are presented as mean ± SD, or mean + SD, indicated in figure legends. Statistical analysis was conducted with GraphPad Prism. Student’s t-test or Fisher’s exact test was used for two-group comparisons. A one-way ANOVA adjusting for multiple comparisons using Tukey’s test procedure was used for multi-group comparisons. P values less than 0.05 were considered statistically significantly different between groups, ∗p < 0.05, ∗∗p < 0.01, ∗∗∗p < 0.001, ∗∗∗∗p < 0.0001, ns, not significant.

## Data Availability

• RNA-seq data related to this study have been deposited at GEO and are publicly available as of the date of publication. Accession numbers are listed in the key resources table. • Any additional information required to reanalyze the data reported in this paper is available from the lead contact upon request. • This paper does not report original codes.
