# Supplementary material for: Interferon hyperactivity impairs cardiogenesis in Down syndrome via downregulation of canonical Wnt signaling
Source: iScience. 2023 Jun 5;26(7):107012. doi: 10.1016/j.isci.2023.107012 (PMC10285545; doi:10.1016/j.isci.2023.107012)
Supplement: Document S1. Figures S1–S10 and Tables S1–S3 [file mmc1.pdf]

iScience, Volume *26*

Supplemental information

Interferon hyperactivity impairs cardiogenesis in Down syndrome via downregulation of canonical Wnt signaling

Congwu Chi, Walter E. Knight, Andrew S. Riching, Zhen Zhang, Roubina Tatavosian, Yonghua Zhuang, Radu Moldovan, Angela L. Rachubinski, Dexiang Gao, Hongyan Xu, Joaquin M. Espinosa, and Kunhua Song

Supplemental Figures and Legends

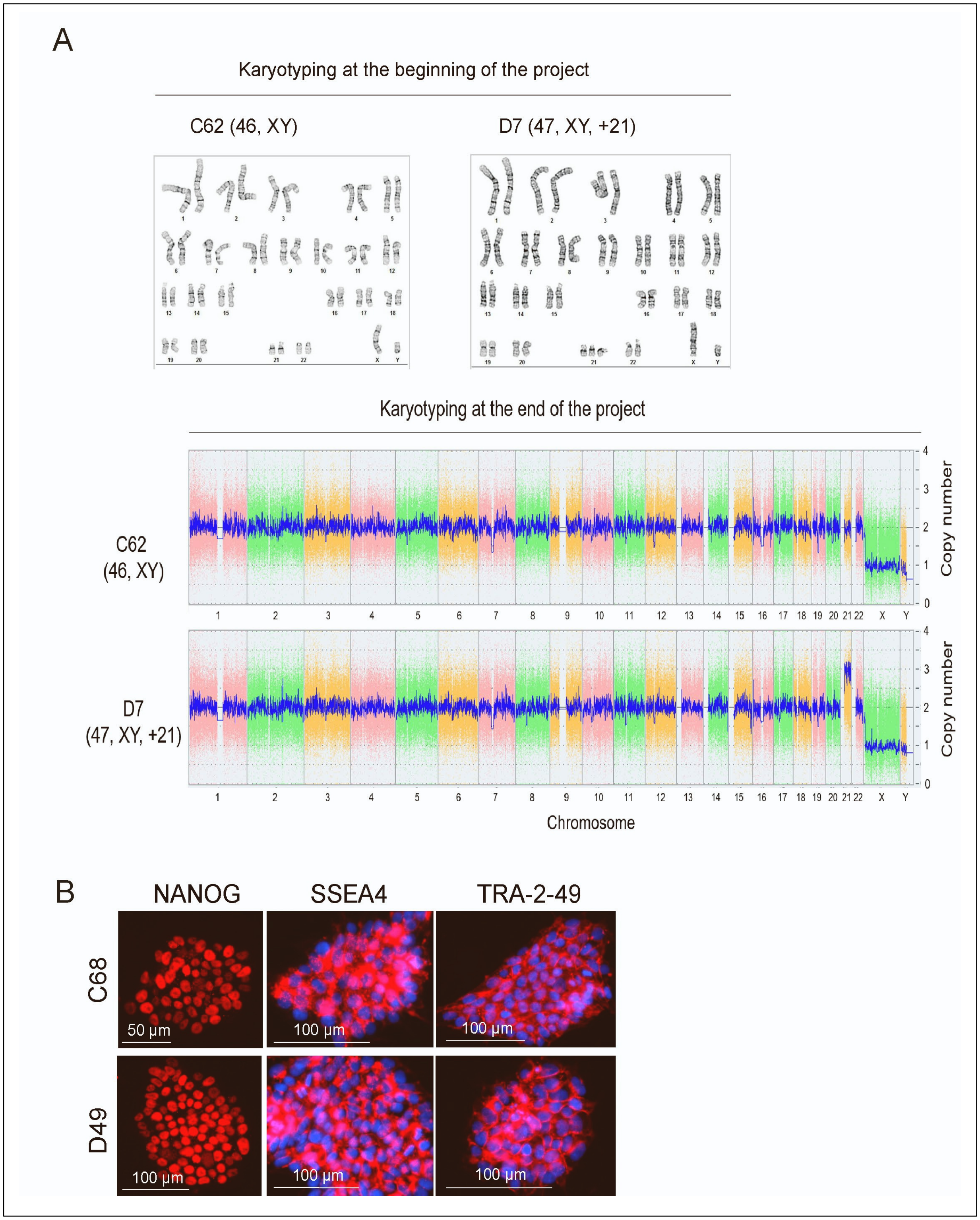

Figure S1. Characterization of human control and DS/CHD iPSCs. Related to **Figure 1.** (**A**) Representative karyotyping images. Control iPSCs in this study had normal (46, XY) karyotype, while DS/CHD iPSCs had T21 (47, XY, +21) karyotype. Initially, T21 in iPSC lines was confirmed by G-banding karyotyping (top panel), assayed at *WiCell* Research Institute and Molecular Pathology Shared Resource Cytogenetics at the University of Colorado. Karyotypic analysis of iPSC lines with the highest passage numbers in this study was performed using the KaryoStat+ ^{TM} Assay (Thermo Fisher Scientific, bottom panel). (**B**) Expression of pluripotency markers NANOG, SSEA4, and TRA-2-49 in control and DS/CHD iPSC colonies. DNA was stained in blue. Scar bars are shown on individual panels.

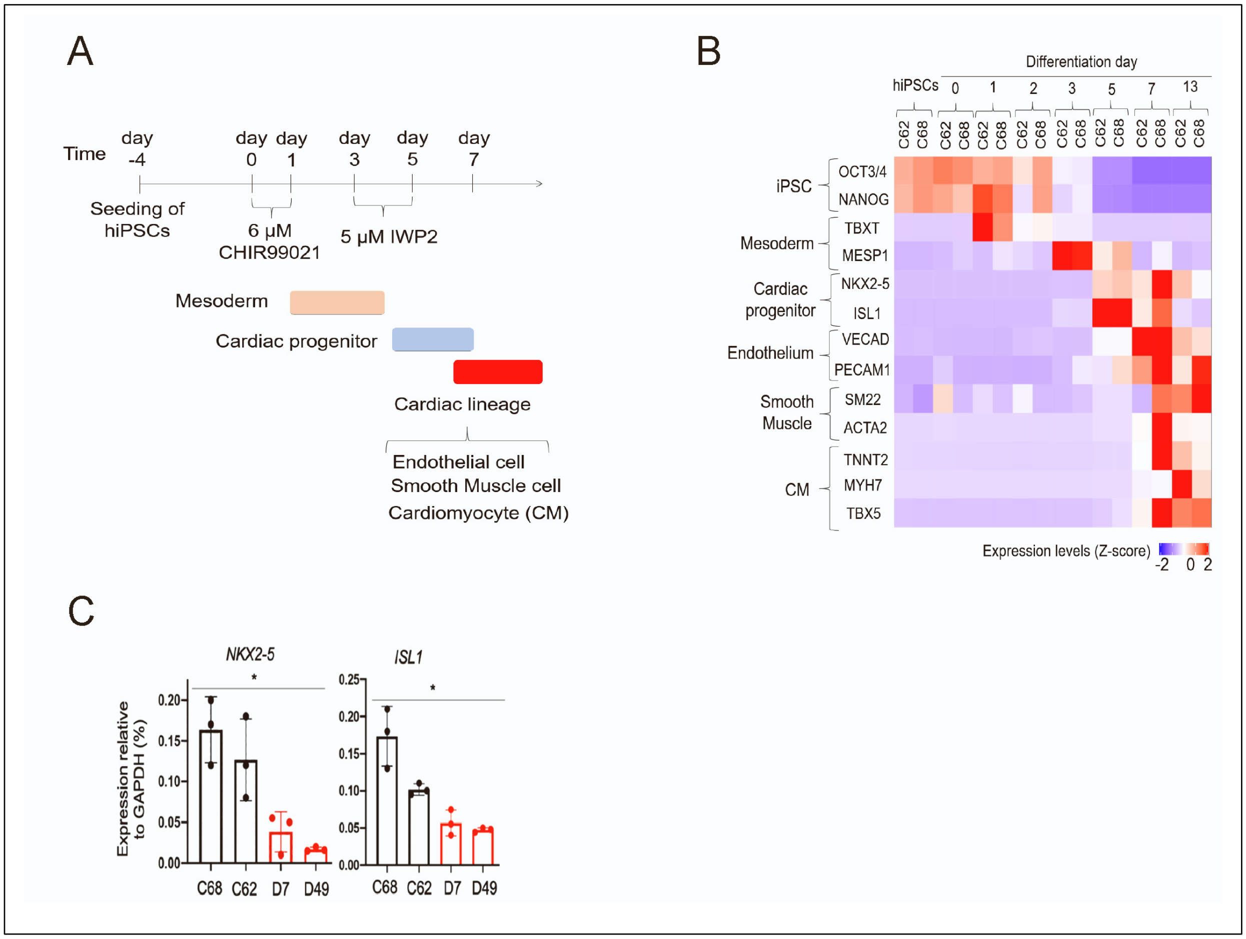

Figure S2. Differentiation of control and DS/CHD iPSCs into cardiac cells using a monolayer-based protocol. Related to **Figure 1.** (**A**) Schematic indicating the protocol for cardiac differentiation of iPSCs and timeline for appearance of lineage cells. (**B**) Expression of pluripotency and lineage markers in control iPSCs and on indicated time (day) post-induction of differentiation. (**C**) Expression of *NKX2-5* and *ISL1* on day 7. Each dot represents an independent experiment. Data are presented as mean with ± SD. *$p < 0.05$ (controls vs DS/CHD), Ordinary one-way ANOVA.

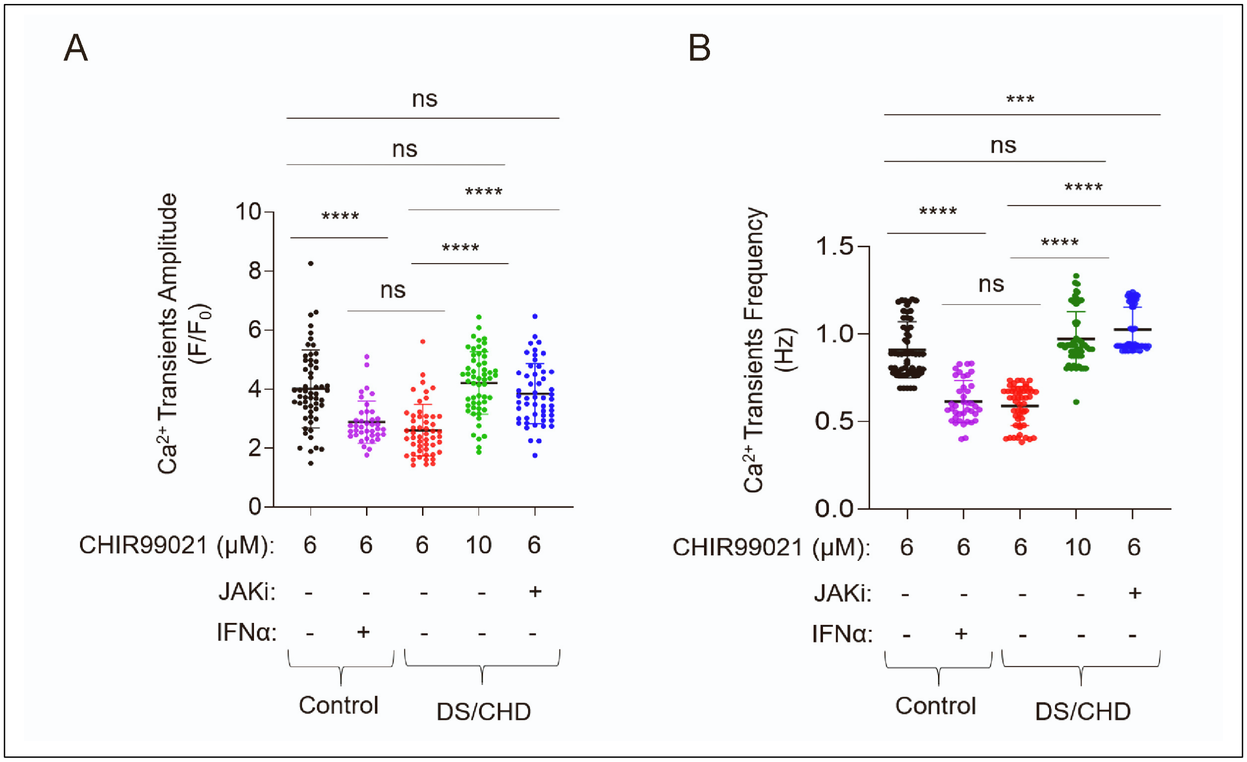

Figure S3. Analysis of Ca^{2+} transients of control and DS/CHD iPSC-CMs on day 13. Related to **Figures 1, 2,** and **3.** Ca^{2+} transients were measured with the genetically encoded Ca^{2+} indicator GCaMP6f (*80*). Spontaneous cytosolic Ca^{2+} signals in cells differentiated from 3 control (C42, C62, and C68) and 3 DS/CHD (D7, D19, and D49) iPSC lines infected with an adenoviral vector encoding GCaMP6f were imaged using a confocal microscope. Fluorescence intensity (F) was normalized to baseline F_0. Amplitude and frequency of Ca^{2+} transients are shown in **A** and **B**, respectively. N = ~10 beating clusters per iPSC line were analyzed. Each filled circle represents an individual cluster. ***p<0.001, ****p<0.0001, ns, not significant, One-way ANOVA.

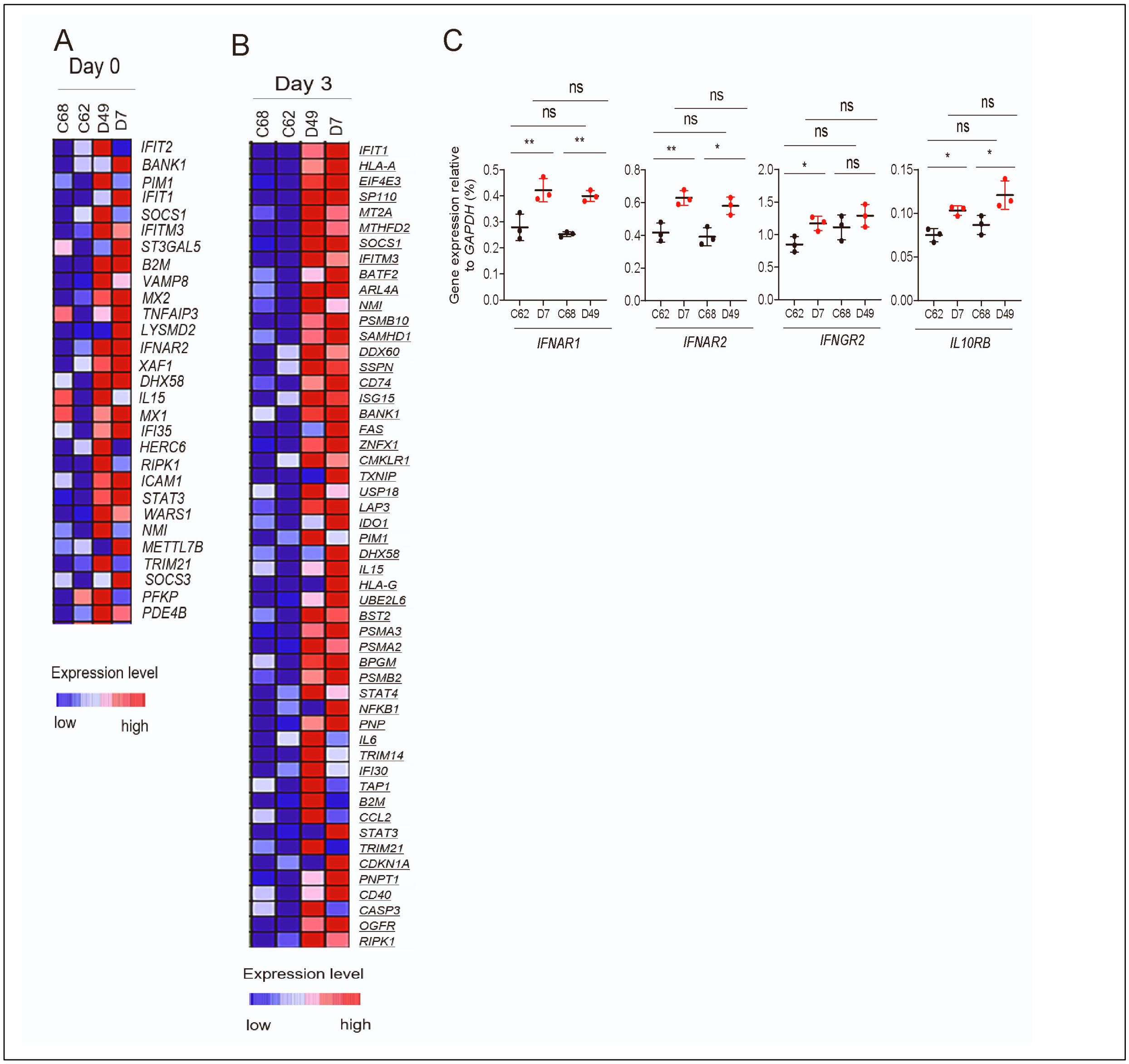

Figure S4. Expression of genes enriched in IFN signaling in cells differentiated from control and DS/CHD iPSCs on days 0 and 3 post-induction of differentiation. Related to **Figure 1.** (**A-B**) Heat map for IFN signaling genes significantly upregulated in DS/CHD cells, compared to control cells. Gene expression levels rang from red (high expression), pink (moderate), light blue (low) to dark blue (lowest expression). (**C**) Expression of IFNR genes on HSA21 on day 3 during cardiac differentiation of control (C62 and C68) and DS/CHD (D7 and D49) iPSCs were analyzed by qPCR. N = 3 independent inductions for each iPSC lines. Each filled circle represents one independent induction of differentiation. Data are presented as mean ± SD. *p<0.05, **p<0.01, ns, not significant, One-way ANOVA.

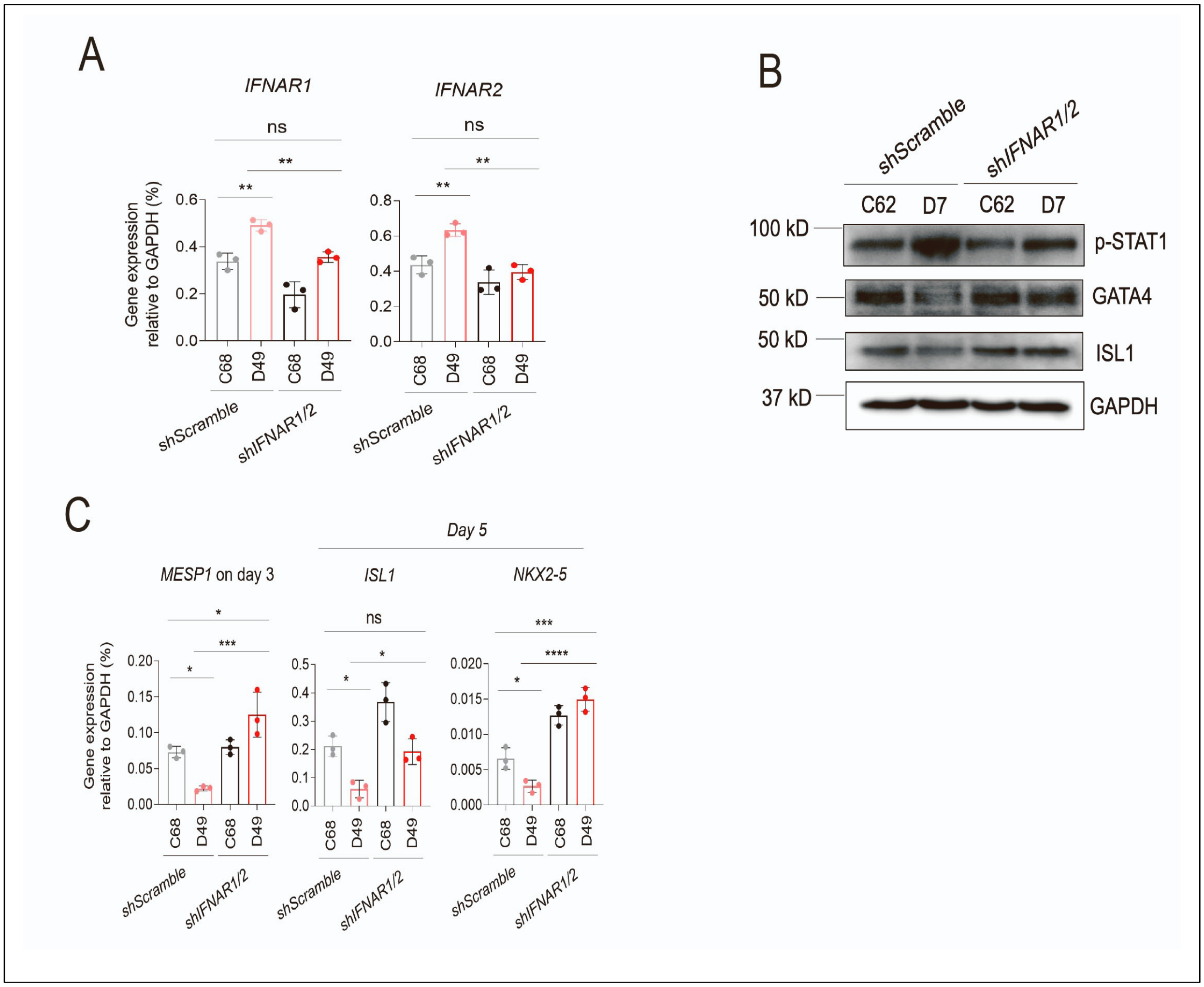

Figure S5. Knockdown (KD) of *IFNAR1* and *IFNAR2* on HSA21 that encode IFN receptors ameliorates cardiac differentiation of DS/CHD iPSCs. Related to **Figure 2.** (**A**) KD of *IFNAR1* and *IFNAR2* in iPSCs using short hairpin RNAs (shRNA). The efficiency of KD was evaluated using qPCR on differentiation day 3. Data are presented as mean ± SD. **p<0.01, ns, not significant, One-way ANOVA. (**B**) Immunoblotting analysis of indicated proteins on differentiation day 7. (**C**) qPCR analysis of gene expression in iPSC-derived cells on differentiation day 3 and day 5. Three independent inductions were performed for each line. Data are presented as mean ± SD. *p<0.05, ***p<0.001, ****p<0.0001, ns, not significant, One-way ANOVA.

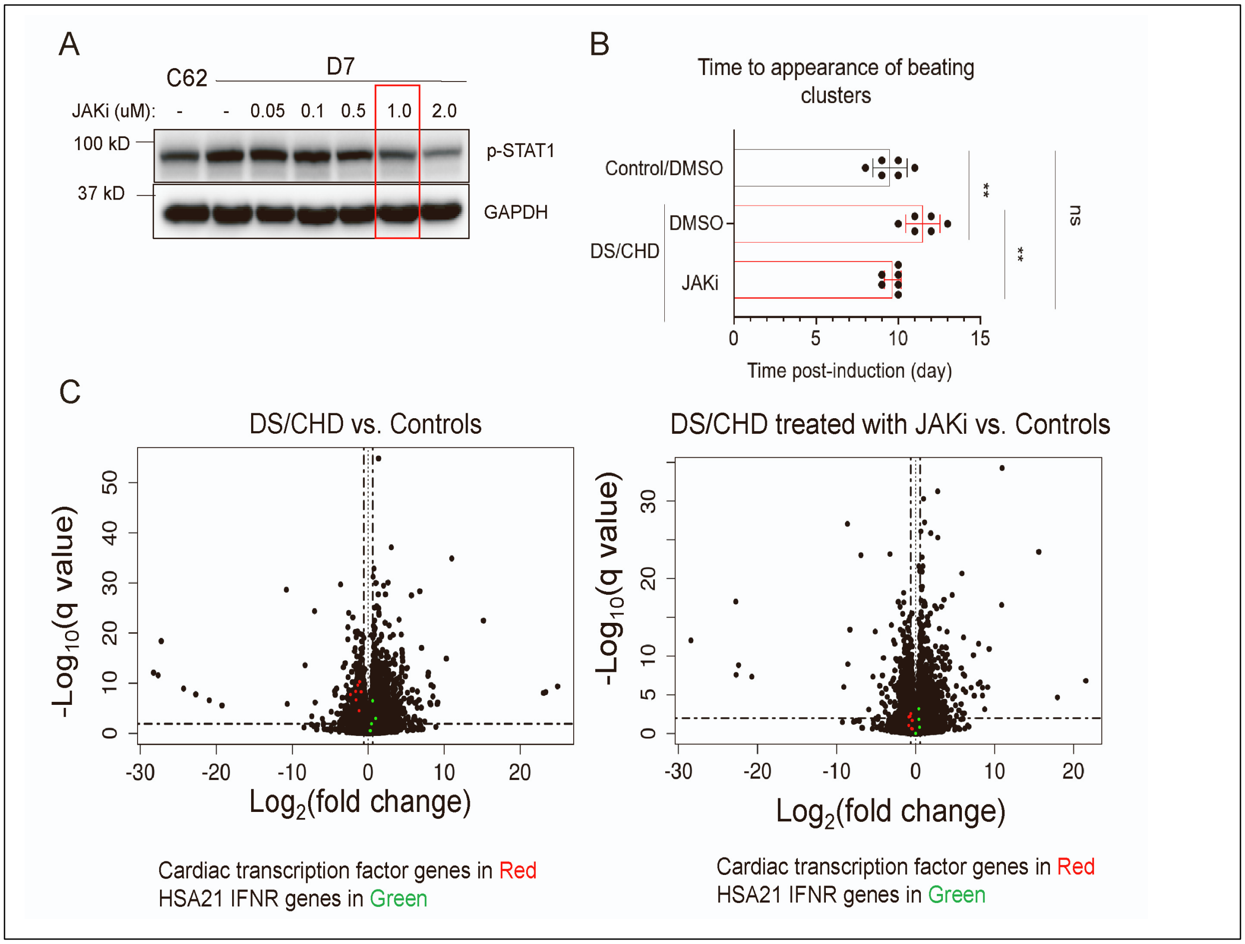

Figure S6. Inhibition of IFN signaling by JAKi ameliorates cardiac gene programming during cardiac differentiation of DS/CHD iPSCs. Related to **Figure 2.** (**A**) Immunoblotting for p-STAT1 and GAPDH on differentiation day 3. DS/CHD iPSCs were treated with various concentration of JAKi from day 0 to day 3. (**B**) Time to appearance of beating clusters in control or DS/CHD cultures post-induction. Controls (C62 and C68) and DS/CHD (D7 and D49) cultures were treated with DMSO or 1μM JAKi from day 0 to day 3. Each filled circle represents one independent experiment, with 3 experiments for each iPSC line. Data are presented as mean ± SD. **p < 0.01, ns, not significant, ordinary one-way ANOVA. (**C**) Volcano plots of up- and downregulated genes in cells differentiated from DS/CHD iPSCs treated with or without JAKi on day 13, compared to control cells. Genes encoding transcription factors involved in cardiac development are in red, such as *TBX20, IRX4, MEF2A, HAND2, HEY2*. Four filled circles in green are 4 IFN receptor genes on HSA21, *IFNAR1*, *IFNAR2*, *IFNGR2*, and *IL10RB*. Note: JAKi treatment significantly restored cardiac gene expression, but did not affect the expression of HSA21 IFNR genes.

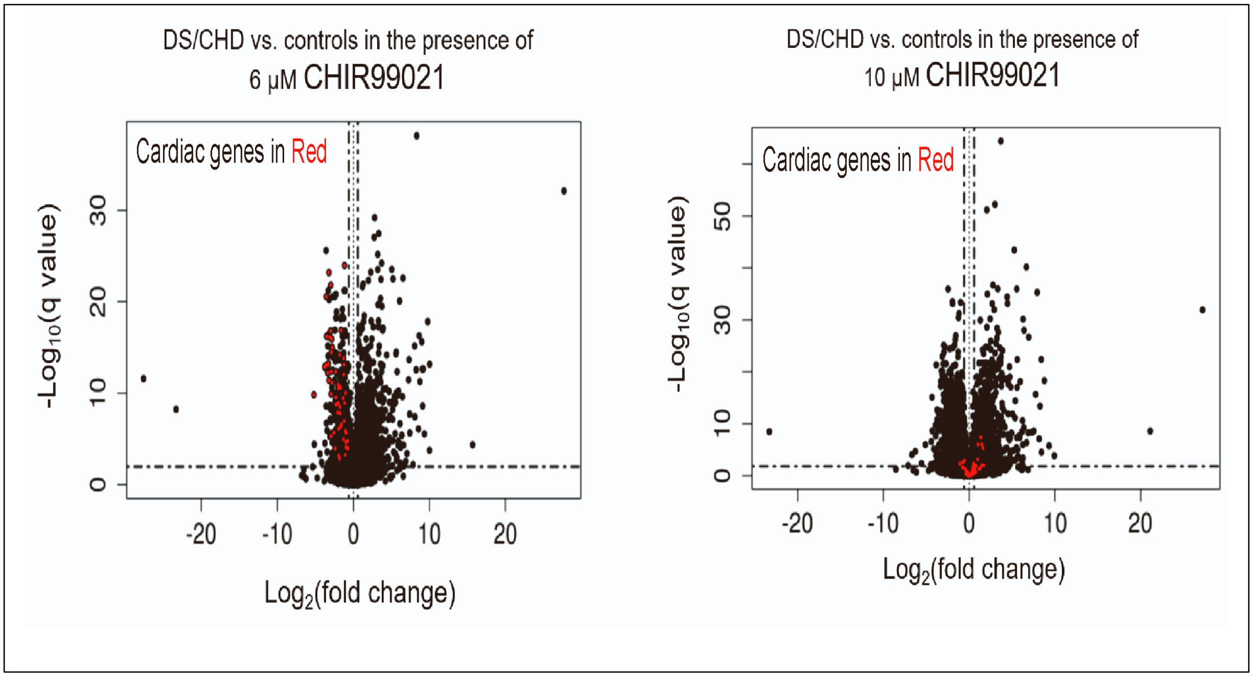

Figure S7. Volcano plots of up- and downregulated genes in cells differentiated from DS/CHD iPSCs treated with 6 μM or 10 μM CHIR99021, compared to control cells. Related to **Figure 3.** Genes involved in cardiac development are in red. These genes are *NKX2-6, TNNC1, ADAMTS6, HSPB7, GATA5, ISL1, SLC8A1, MYBPC3, RBM24, MYL3, BMP2, MEF2A, HEG1, HCN4, NFATC1, TNNI1, BVES, SYNPO2L, ACTN2, PKP2, KDM6A, ACTC1, S1PR1, LDB3, NKX2-5, MYO18B, TBX2, TNNC1, TGFB2, POPDC2, PDLIM5, SMARCD3, TBX20, MEF2C, PDGFRA, CFC1, NRP2, HAND2, NRP1, SYPL2, CACNA1C, TNNT2, HAS2, CSRP3, POPDC3, NPPA, ANKRD1, APLNR, SMYD1, MYLK3, WNT2, RARB, WNT5A, MYL7, RXRG, ADPRHL1, MYH7, TBX5, MYOCD*. Note: increasing the activity of the WNT signaling dramatically restored the expression of these cardiac genes during cardiac differentiation of DS/CHD iPSCs.

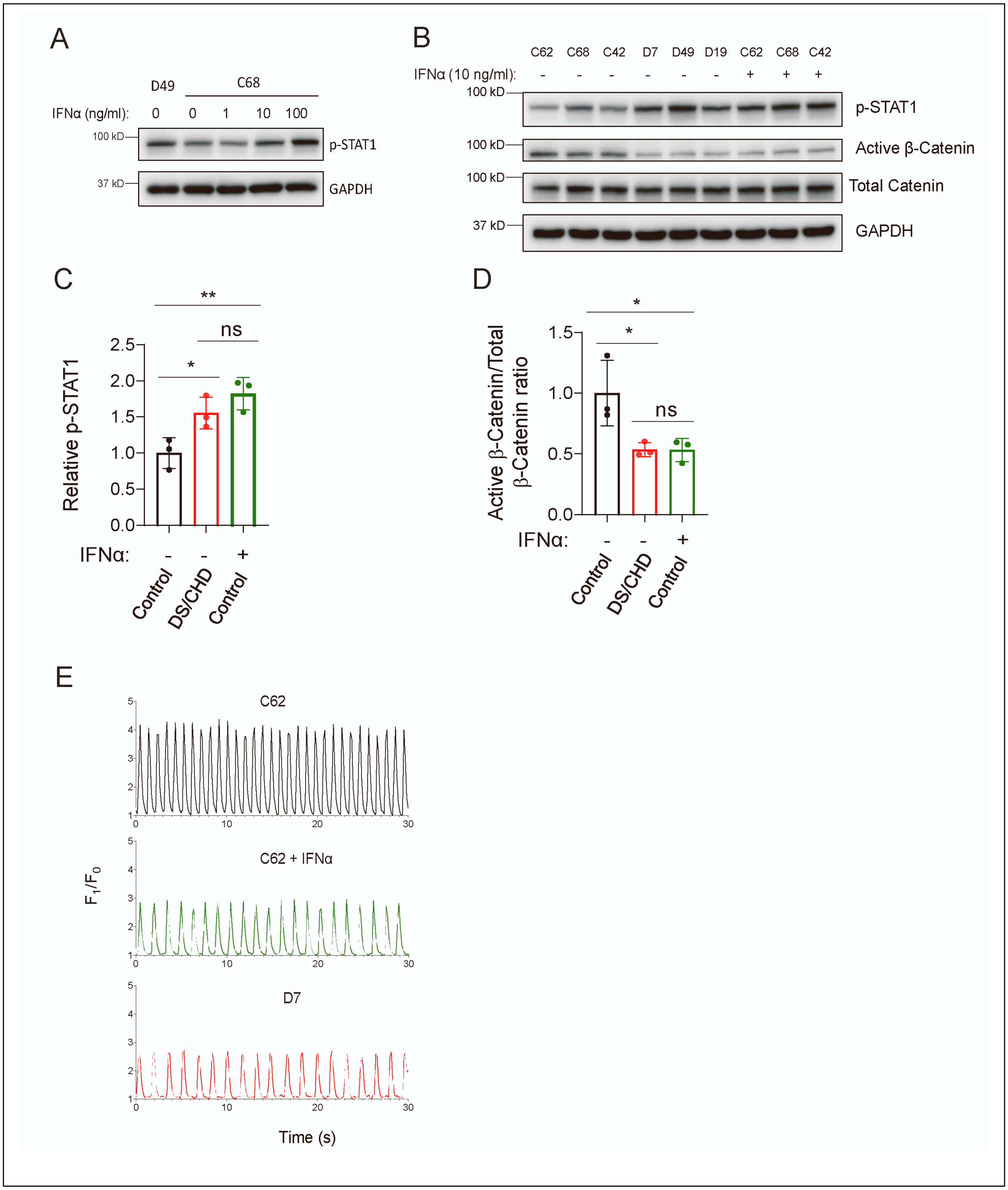

Figure S8. IFNα Treatment of control iPSCs during cardiac differentiation. Related to **Figure 3**. (**A**) Immunoblotting for p-STAT1 and GAPDH on differentiation day 3. Control iPSCs were treated with various concentrations of IFNα from day 0 to day 3 to optimize the IFN concentration

to activate IFN signaling. Treatment of control cells with 10 ng/ml IFNα activated the IFN pathways to a level comparable to DS/CHD cells. (**B**) Three control (C62, C68, C42) iPSC lines were treated with or without 10 ng/ml IFNα from day 0 to day 3. Indicated proteins were analyzed by immunoblotting. (**C-D**) Quantification of relative p-STAT1 levels and the ratio of active β-Catenin to total β-Catenin is presented in **C** and **D**, respectively. Each filled circle represents an individual iPSC line. Data are presented as mean ± SD. *p<0.05, **p<0.01, ns, not significant, One-way ANOVA. (**E**) Representative traces of normalized GCaMP6f signal intensity showing Ca^{2+} transients in control iPSC-CMs (C68), DS/CHD iPSC-CMs (D49), and control cultures treated with 10 ng/ml IFNα from day 0 to day 13. Ca^{2+} signals were imaged on day 13.

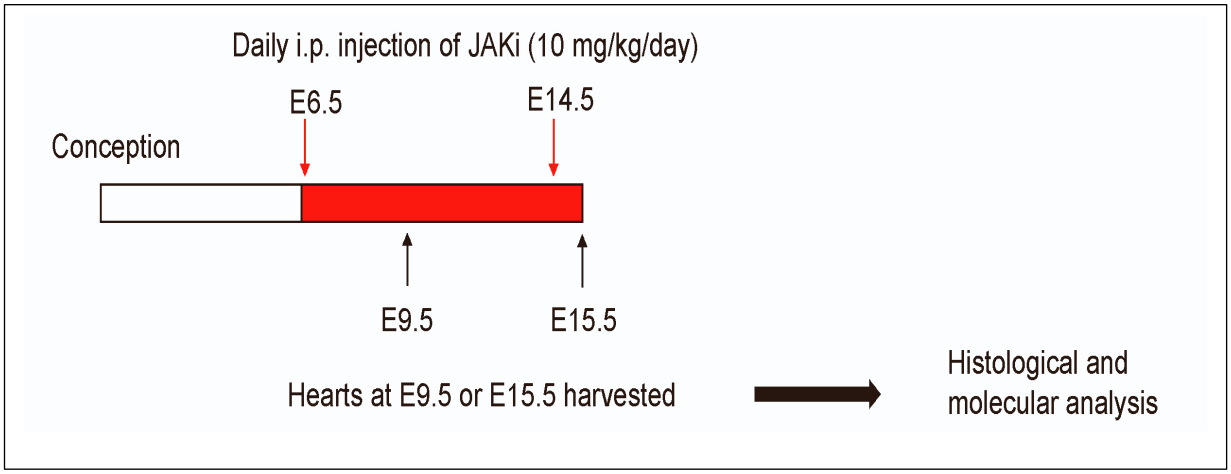

Figure S9. A schematic diagram of JAKi treatment. Related to **Figure 4**. Pregnant mice were daily treated with the JAKi (10 mg/kg body weight/day, i.p. injection) beginning on day 6.5 post-conception. Hearts were harvested at E9.5 or E15.5 for analysis.

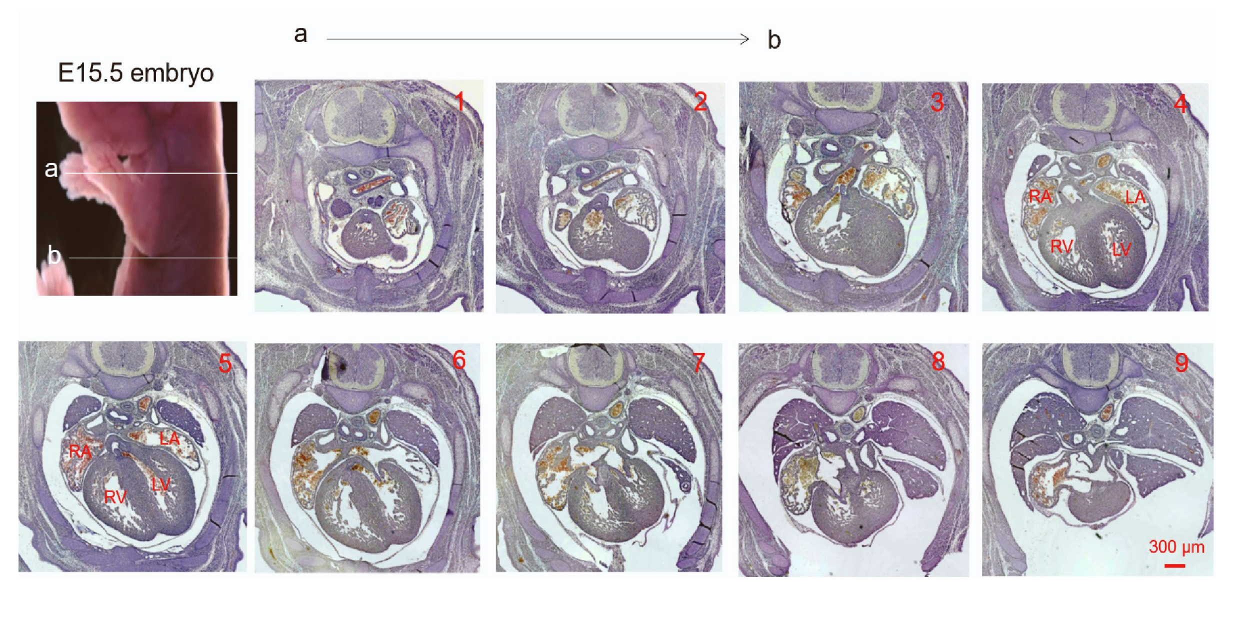

Figure S10. Representative hematoxylin and eosin-stained sections of a WT mouse heart at embryonic day (E)15.5. Related to **Figure 4.** Serial sagittal sections of the developing heart at an interval of 20 μm were cut through the entire heart along the direction of **a** to **b**. Sections with septal defects identified using the EVOS FL Cell Imaging System were stained with hematoxylin and eosin. RA: right atrium; LA: left atrium; RV: right ventricle; LV: left ventricle. Scale bar, 300 μm.

Supplemental Tables

Table S1. iPSC lines are derived from individuals with D21 or T21 and cooccurring congenital heart defects (CHDs). To avoid the possibility that iPSC line variations interfere with phenotypes, we generated iPSC lines from three pairs of human subjects with D21 or T21 and cooccurring congenital heart defects (C62 and D7, C68 and D49, C42 and D19). Three healthy donors are C62, C68, and C42. Three individuals with DS and CHDs are D7, D49, and D19. Each pair is gender- and age-matched. Karyotyping of all iPSC lines was conducted at the project's beginning and end. M, male. D21, disomy of chromosome 21. T21, trisomy of chromosome 21. ASD, atrial septal defect. VSD, ventricular septal defect. PFO, patent foramen ovale. PDA, patent ductus arteriosus. Related to **Figures 1-3**.

ID	Gender	Age (year)	Cohort	Heart conditions	Karyotype	
					At the beginning of the project	At the end of the project
C62	M	27.1	D21	None	46, XY	46, XY
D7	M	30.3	T21	ASD	47, XY, +21	47, XY, +21
C68	M	39	D21	None	46, XY	46, XY
D49	M	39.1	T21	VSD	47, XY, +21	47, XY, +21
C42	M	6.1	D21	None	46, XY	46, XY
D19	M	6.2	T21	PFO/PDA	47, XY, +21	47, XY, +21

Table S2. All gene sets enriched in DS/CHD cells during cardiac differentiation of iPSCs, with a p-value < 0.05. Gene sets involved in the inflammatory response are highlighted in bold. Related to **Figure 1** and **Figure S4**.

	GS	Size	ES	NES	NOM p-value	FDR q-value
Day 0	HALLMARK_TGF_BETA_SIGNALING	49	0.60	2.06	0.000	0.000
	HALLMARK_TNFA_SIGNALING_VIA_NFKB	154	0.46	1.89	0.000	0.004
	HALLMARK_PROTEIN_SECRETION	89	0.49	1.85	0.000	0.002
	HALLMARK_WNT_BETA_CATENIN_SIGNALING	31	0.57	1.77	0.002	0.007
	HALLMARK_CHOLESTEROL_HOMEOSTASIS	66	0.45	1.62	0.005	0.030
	HALLMARK_MTORC1_SIGNALING	181	0.38	1.60	0.000	0.030
	HALLMARK_APOPTOSIS	127	0.39	1.55	0.003	0.039
	HALLMARK_ANDROGEN_RESPONSE	84	0.40	1.50	0.011	0.058
	**HALLMARK_INTERFERON_GAMMA_RESPONSE**	**123**	**0.35**	**1.40**	**0.016**	**0.123**
	HALLMARK_HYPOXIA	172	0.33	1.39	0.007	0.113
	HALLMARK_COAGULATION	79	0.37	1.38	0.017	0.114
	HALLMARK_HEDGEHOG_SIGNALING	24	0.47	1.38	0.087	0.107
	HALLMARK_REACTIVE_OXYGEN_SPECIES_PATHWAY	38	0.43	1.34	0.094	0.129

	**HALLMARK_IL2_STAT5_SIGNALING**	**141**	**0.32**	**1.30**	**0.039**	**0.169**
	HALLMARK_MYOGENESIS	127	0.32	1.27	0.047	0.187
Day 3	HALLMARK_MYC_TARGETS_V1	200	0.57	2.17	0.000	0.001
	HALLMARK_MYC_TARGETS_V2	58	0.60	1.94	0.000	0.001
	HALLMARK_E2F_TARGETS	198	0.50	1.92	0.000	0.001
	HALLMARK_ALLOGRAFT_REJECTION	139	0.49	1.81	0.000	0.003
	HALLMARK_OXIDATIVE_PHOSPHORYLATION	199	0.45	1.75	0.000	0.009
	HALLMARK_MTORC1_SIGNALING	198	0.44	1.68	0.001	0.015
	HALLMARK_G2M_CHECKPOINT	197	0.42	1.63	0.002	0.022
	HALLMARK_HYPOXIA	187	0.40	1.54	0.000	0.049
	HALLMARK_PANCREAS_BETA_CELLS	29	0.55	1.53	0.042	0.047
	HALLMARK_APOPTOSIS	142	0.41	1.53	0.007	0.045
	HALLMARK_UNFOLDED_PROTEIN_RESPONSE	112	0.43	1.53	0.010	0.042
	HALLMARK_EPITHELIAL_MESENCHYMAL_TRANSITION	185	0.39	1.47	0.011	0.063
	HALLMARK_KRAS_SIGNALING_UP	145	0.39	1.44	0.019	0.081
	**HALLMARK_TNFA_SIGNALING_VIA_NFKB**	**175**	**0.38**	**1.43**	**0.019**	**0.079**
	HALLMARK_UV_RESPONSE_UP	147	0.39	1.43	0.016	0.076
	**HALLMARK_INTERFERON_GAMMA_RESPONSE**	**154**	**0.39**	**1.42**	**0.013**	**0.073**

Table S3. qPCR primers. Related to **Figures 2, S2, S4,** and **S5**.

**Primer IDs**	**Sequences (5' -> 3")**	**Genes**
hIFNAR1-F	CACTTCTTCATGGTATGAGGTTGACT	Human *IFNAR1*
hIFNAR1-R	ATTGCCTTATCTTCAGCTTCTAAATGT	Human *IFNAR1*
hIFNAR2-F	TCATATGATTCGCCTGATTACACA	Human *IFNAR2*
hIFNAR2-R	TGGTACAATGGAGTGGTTTTTTAATT	Human *IFNAR2*
hIFNGR2-F	GGAAAAGGAGCAAGAAGATGTTCT	Human *IFNGR2*
hIFNGR2-R	AGCTCCGATGGCTTGATCTC	Human *IFNGR2*
hIL10RB-F	GGAATGGAGTGAGCCTGTCTGT	Human *IL10RB*
hIL10RB-R	AAACGCACCACAGCAAGGCGAA	Human *IL10RB*
hMESP1-F	CTGAAGGGCAGGCGATG	Human *MESP1*
hMESP1-R	CCTTGTCACTTGGGCTCC	Human *MESP1*
hISL1-F	GCGGAGTGTAATCAGTATTTGGA	Human *ISL1*
hISL1-R	GCATTTGATCCCGTACAACCT	Human *ISL1*
hNKX2-5-F	CAAGTGTGCGTCTGCCTTT	Human *NKX2-5*
hNKX2-5-R	CAGCTCTTTCTTTTCGGCTCTA	Human *NKX2-5*
hGAPDH-F	CTCTGGTAAGTGGATATTGTTGCC	Human *GAPDH*
hGAPDH-R	AGAGATGATGACCCTTTTGGCTCC	Human *GAPDH*
